# Photographic-Based Optical Evaluation of Tissues and Biomaterials Used for Corneal Surface Repair: A New Easy-Applied Method

**DOI:** 10.1371/journal.pone.0142099

**Published:** 2015-11-13

**Authors:** Miguel Gonzalez-Andrades, Juan de la Cruz Cardona, Ana Maria Ionescu, Charles A. Mosse, Robert A. Brown

**Affiliations:** 1 Tissue Repair & Engineering Centre, University College of London, London, United Kingdom; 2 Tissue Engineering Group, Department of Histology, University of Granada, Granada, Spain; 3 Ophthalmology Department, San Cecilio University Hospital, Granada, Spain; 4 Laboratory of Biomaterials and Optics—Optics Department, University of Granada, Granada, Spain; 5 Optics Department, University College of London, London, United Kingdom; University of Milano-Bicocca, ITALY

## Abstract

**Purpose:**

Tissues and biomaterials used for corneal surface repair require fulfilling specific optical standards prior to implantation in the patient. However, there is not a feasible evaluation method to be applied in clinical or Good Manufacturing Practice settings. In this study, we describe and assess an innovative easy-applied photographic-based method (PBM) for measuring functional optical blurring and transparency in corneal surface grafts.

**Methods:**

Plastic compressed collagen scaffolds (PCCS) and multilayered amniotic membranes (AM) samples were optically and histologically evaluated. Transparency and image blurring measures were obtained by PBM, analyzing photographic images of a standardized band pattern taken through the samples. These measures were compared and correlated to those obtained applying the Inverse Adding-Doubling (IAD) technique, which is the gold standard method.

**Results:**

All the samples used for optical evaluation by PBM or IAD were histological suitable. PCCS samples presented transmittance values higher than 60%, values that increased with increasing wavelength as determined by IAD. The PBM indicated that PCCS had a transparency ratio (TR) value of 80.3±2.8%, with a blurring index (BI) of 50.6±4.2%. TR and BI obtained from the PBM showed a high correlation (ρ>|0.6|) with the diffuse transmittance and the diffuse reflectance, both determined using the IAD (p<0.005). The AM optical properties showed that there was a largely linear relationship between the blurring and the number of amnion layers, with more layers producing greater blurring.

**Conclusions:**

This innovative proposed method represents an easy-applied technique for evaluating transparency and blurriness of tissues and biomaterials used for corneal surface repair.

## Introduction

One of the most common cause of blindness globally is the consequence of corneal pathology[1]. Several procedures have been described to repair the corneal surface not only to reestablish the physical barrier function of the cornea, but also to increase its transparency, and improve visual acuity of the patient. One part of these approaches relies on substrates different to corneal tissue to regenerate the corneal surface. Amnion has been used for that purpose since 1940s, applied as a graft or as a patch over corneal ulcers or defects[2, 3]. However, amnion has the major disadvantage of being biologically variable between donors, not least in transparency. Moreover, amnion is labour intensive to collect and prepare and costly to screen[4]. Thus, different research groups are trying to generate scaffolds that could substitute for corneal matrix without resorting to amnion, using a range of natural biomaterials from fibrin[5] to fibrin-agarose[6] and collagen[7]. However, even where these materials are produced by standardized manufacture, careful control is needed not least to assess their optical properties prior to implantation. Hence, we need to identify suitable corneal replacements materials, or amnion patches, based on critical and objective optical evaluation.

Collagen hydrogels, like plastic compressed collagen scaffolds (PCCS), are an ideal alternative material for resurfacing cornea and for corneal epithelial cell delivery. Such collagen supported corneal limbal cell layers differentiate and produce an appropriate basement membrane under cell-cell and cell matrix interactions in culture[7]. Similar tissue and cell delivery technology has been proposed for a number of tissue engineering applications, including skin[8], cornea [7, 9, 10], bone[11], bladder[12], spinal cord [13] and peripheral nerve repair, and neovascularization angiotherapy strategies [14, 15]. The current use of biomaterials for drug delivery and anterior grafts for ocular surface repair is increasing, including limbal stem cell deficiency (LSCD) [16]. In this context, cultured limbal epithelial transplantation (CLET) is one of the most successful cell therapies in use [17]. CLET is based on the culture of limbal epithelial stem cells (LESC) on a range of substrate materials to promote cell growth under good manufacturing practices (GMP). Many researchers use substrates based on fibrin or amniotic membrane[18–20]. Nevertheless, one of the most important properties of these corneal substitutes must be their transparency. Note that the human cornea is a transparent tissue responsible for the most of the refractive power of the eye, allowing the incoming light to reach the retina. The corneal transparency has been the subject of numerous studies over the years and it is now generally accepted that it depends on the destructive interference of the incoming light that is scattered away and on the absorption of light by the corneal tissue itself[5]. Therefore, an easily applicable method to evaluate the optical properties of the proposed GMP complient substrates is needed.

There are a range of existing methods to evaluate the optical properties of biomaterials or the native cornea[21]. These include direct methods, including those based on fundamental concepts and rules, such as the Bougier-Beer law, the single scattering phase function for thin samples or the effective light penetration depth for slabs. There are also indirect methods that deduce the solution of the inverse scattering problem using a theoretical model light propagation within a medium. These would include the Kubelka-Munk model, the Monte Carlo method or the Inverse Adding-Doubling method (IAD), which is currently the gold-standard method. The disadvantage of these methods is that it is necessary to strictly fulfill experimental conditions dictated by the selected model (specific equipment such as light detectors, spectrophotometers, lasers) that are not always available in biological or clinical laboratories. In some cases, proper measurements of the transmitted/reflected/absorbed/scattered incident light throughout the biotissues cannot be performed and in others assessment of tissue transparency is made by simply placing the sample on top of a reference image and subjectively estimating the similarities with a control sample. All such techniques are observer-dependent and do not provide quantitative, numerical data[22]^,^ [23]^,^ [24]. Consequently, it has become important to the future development of corneal implant materials and scaffolds to develop a simple, practical economical and accurate method, by which biomedical laboratories and GMP facilities can report quantitative targets and establish the limits of biomaterial usefulness in terms of use which are functionally relevant to the patient. In modern society this is commonly the ability to distinguish between key-word letters, despite edge blurring. Importantly, we currently do not really have a good objective measure of the levels of transparency reduction and blurring, due to substrate materials, corneal grafts or amnion patches, which are tolerable or intolerable for patients. Therefore, the main goal of this study was to describe and assess a quantitative measure of functional optical blurring and transparency, based on simple economical technology, which could serve these defining functions. The method described is based on photographic images taken through the test material (PCCS) to evaluate optical properties likely to be important to patient function. Finally, we have applied this new method to that material which is most widely used in corneal surface repair, amniotic membrane, to evaluate its feasibility for application in clinically related GMP processes.

## Materials and Methods

### PCCS construction and amniotic membrane preparation

PCCS (n = 3) of 1 layer of 4 ml of collagen solution were generated following the method previously described by Hadjipanayi *et al*.[25]. Collagen gels were prepared by sodium hydroxide (Sigma-Aldrich, Dorset, UK) neutralization of 13 ml (80%) of sterile Nutragen® collagen solution (Advanced BioMatrix, Inc.; San Diego, California), 1.5 ml (10%) of 10x Minimum Essential Medium (Invitrogen Ltd, Paisley, UK) and 1.5 ml (10%) of Dulbecco's Modified Eagle Medium (Invitrogen Ltd, Paisley, UK). 4 ml collagen gels were cast in 22 mm diameter circular moulds for fibrillogenesis at 37°C. Then, the plastic compression was performed using filter paper (Fisherbrand QL 100, 270mm; Code FBS9033) and chromatography paper (Whatman chromatography paper,1 reel; Cat no. 3001652) over the collagen gel. Afterwards, samples were evaluated immediately. Amniotic membrane (AM) samples were obtained from the remnants recovered after ocular surface reconstruction surgery performed in the San Cecilio University Hospital, Granada, Spain, where AM was employed. Written informed consents from the donors were obtained for the use of these samples for clinical and research purpose. The institutional review board waived the need for a specific consent for this project for being anonymous remnant tissues after surgery. AM was folded several times and placed into a petri dish for analysis. 4 versions of multilayered amnion were prepared using between 1 and 4 layers after folding. The thickness of each sample was measured using a Nikon Eclipse 90i light microscope (Nikon Instruments Inc., USA).

### Histology

AM samples and PCCS were histologically evaluated. For light microscopy, samples were fixed in 4% paraformaldehyde for 20 minutes, dehydrated in an ethanol series, and embedded in paraffin. Full depth 5-micron sections were stained with Sirious red and Hematoxilin and Eosin, and examined with Nikon Eclipse 90i light microscope (Nikon Instruments Inc., USA).

### Determination of the optical properties by the photographic-based method (PBM): Transparency and blurriness evaluation

**Fig 1:**
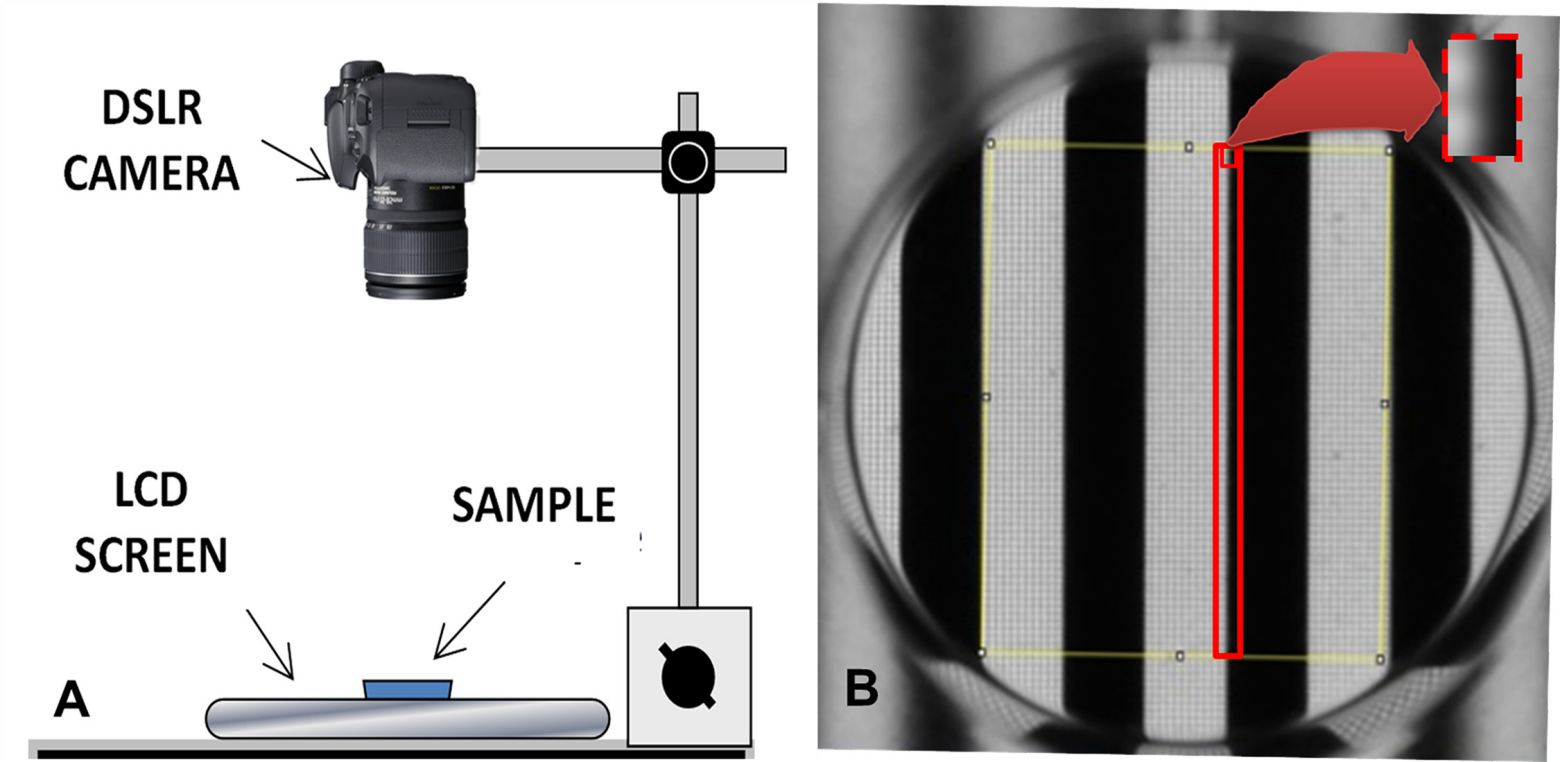
Elements used for performing the photographic-based method. A: Diagram of the camera and the LCD screen for measuring the transparency of the sample. B: Black-white stripe pattern (3 mm-width stripe each) observed through the petri dish, where a rectangle area (red rectangle in image) of 354 microns-width and 19000 microns-height was selected to perform a plot profile for the blurring assay. The red dot rectangle magnifies this in more detail.

Transparency and image blurring measures were obtained by analyzing photographic images of a standardized band pattern taken through the sample membranes. A black-white stripe pattern (3 mm-width stripe each) was displayed on a12.1 inch (1024 x 768 pixels, 41.65 pixels/cm) TFT laptop screen (Lenovo ThinkPad X61, New South Wales, Australia) with 100% brilliance intensity. A petri dish containing the test material (with a known number of layers) was placed over this pattern, as shown in Fig 1. Baseline comparator measures were made using an identical empty petri dish. The distance between the computer screen (standard pattern) and the camera objective was 32cm. 11 pictures of each sample, in RAW format (4288x2848 pixels),were taken using ‘interval shooting’ mode (a 1 second of interval) using a Digital Single Lens Reflex (DSLR) camera (12,3MPxCMOS sensor -23.6x15.8 mm-, Nikon D5000,Nikon Co, Japan) and a 55-200mm VR objective, set at 200mm. All the parameters related to optical magnification and camera sensor were taken into account to obtain a higher number of pixels, and their associated values, from the black white edge of the stripe pattern. In each case, the first image taken was discarded. Prior to digital image acquisition, the photographic camera was white balance calibrated using a white/gray card (QP101 Calibration Card, QPCard, Sweden).The camera mode was set to manual, and all shooting parameters (shutter speed 1/160, aperture F5.6 and ISO800) were maintained constant throughout the whole process, in order to ensure proper comparison between the samples. All measurements were performed by the same user in order to avoid experimenter-related differences.

The aim of the blurring analysis was to produce a single number that could be used as an index by which to objectively compare different materials or corneal tissues. ImageJ software was used to analyze the sharpness of the stripes (ie. the functional blurring of the test specimen). This software can be used to define a rectangle in which it will compute the average intensity for each column of pixels. It will then plot a profile of the average gray scale intensity of each column (y scale) versus the column’s distance in pixels across the rectangle. The rectangles were constructed across a specific length of the black white edge of the stripe pattern with the aim of quantifying loss of this initially sharp edge as shown in Fig 1 (ie. image blurring). Each rectangle was 0.35 mm wide x 19 mm long, corresponding to 26 x 1400 pixels of the picture such that the profile from a perfectly sharp image (ie the empty petri dish) would be a vertical step whilst increasingly blurring materials would convert this into a more sloping profile. The profiles were next scaled so that the step height between the centres of the black regions and the centres of the white regions were the same. The standard deviation (SD) of the 26 points along the profile indicated the sharpness of the contrast between the black and white regions. The expression proposed for calculating the “Blurring Index” (BI) was the following:

$$BI=1-\frac{{SD}_{sample\;profile}}{{SD}_{empty\;Petri\;dish}}$$

where *SD*_*sample profile*_ is the standard deviation obtained for a profile with the sample placed on top of the Petri dish and *SD*_*empty Petri dish*_ is the standard deviation obtained for a profile with no sample placed on top of the Petri dish. In effect, blurred image produce a gently sloping profile where many of the points are close to the mean (value of BI close to 1). By contrast, a perfectly sharp image (minimally blurring material) would leave all the points either at the top or at the bottom of the scale, at the maximum distance from the mean (BI close to 0).

**Fig 2:**
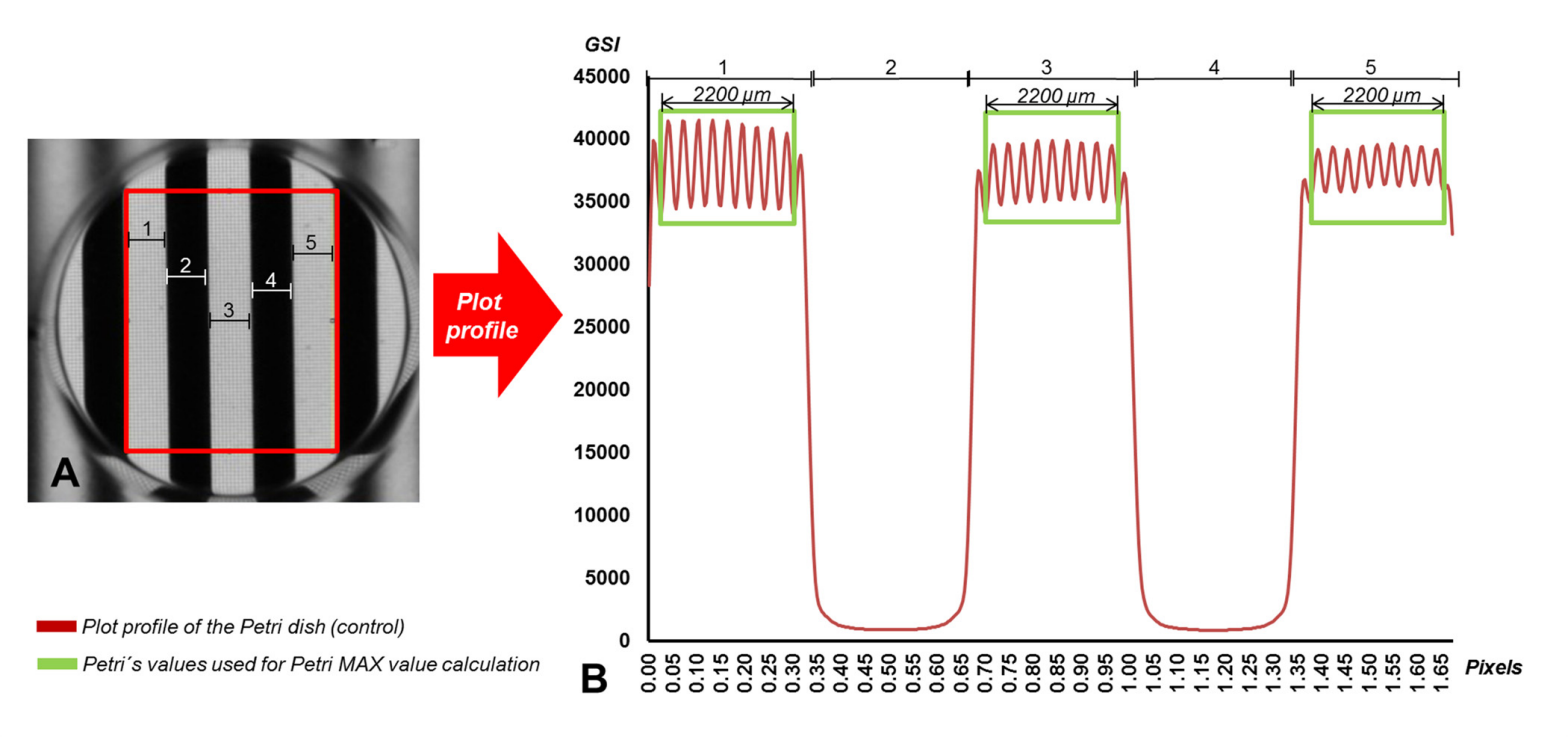
Transparency assay of the control (petri dish only without sample). A: Black-white stripe pattern (3 mm-width stripe each) observed through the petri dish, where a rectangle area (red rectangle) of 19mm-height and 15mm-wide was selected to perform a plot profile for the transparency assay. B: In the plot profile obtained, the mean value of the points found in the 2200 microns in the center of each of the three white stripes (green rectangle) found in the previous selected area was calculated to obtain the MAX value of the petri dish.

The transparency assay was performed as follow. A rectangular area (19mm-height, 15mm wide) was selected to perform a plot profile of each sample using ImageJ Software (Fig 2). All case samples were triplicated and averaged. The mean value of the points found in the 2200 microns in the center of each of the three white stripes found in the previous selected area, was calculated (the MAX value). Then, the transparency ratio (TR) was calculated as

$$TR=\frac{{MAX}_{Sample}}{{MAX}_{Petri\;dish}}$$

Where the *MAX*_*Sample*_ is the MAX value of each sample and the *MAX*_*Petri dish*_ is the MAX value obtained in the petri dish control. Finally, a comparison between the different TRs of each kind of sample was performed.

### Determination of the optical properties by the Comparator Method: Inverse Adding-Doubling (IAD) technique

IAD technique[26] was used in this study to determine the reflectance, transmittance, scattering and absorption properties of PCCS constructs, to allow detailed comparison and correlation with the photographic-based blurring evaluation developed here. Total diffuse reflection and total diffuse transmission measurements were performed on each sample. Total diffuse reflection measurements were made using a 158.2mm-diameter integrating sphere (Oriel, model 70674. Newport. USA) with an 10mm-diameter detector port and a 7mm-diameter sample port with a baffle between ports and a 14mm diameter entrance port. Measurements were performed on the PCCS at wavelengths of 457.9nm, 488nm and 514.5nm from an argon ion laser (Stellar-Pro-L Model, Modu-Laser, USA) and 632.8nm from a He-Ne laser (30564 Model, Research Electro-Optics, USA). Maximum laser output power was 1000mW±5% for the argon laser and 12mW±0.2% for the He-Ne laser and the diameter of both laser beams was 2mm.

**Fig 3:**
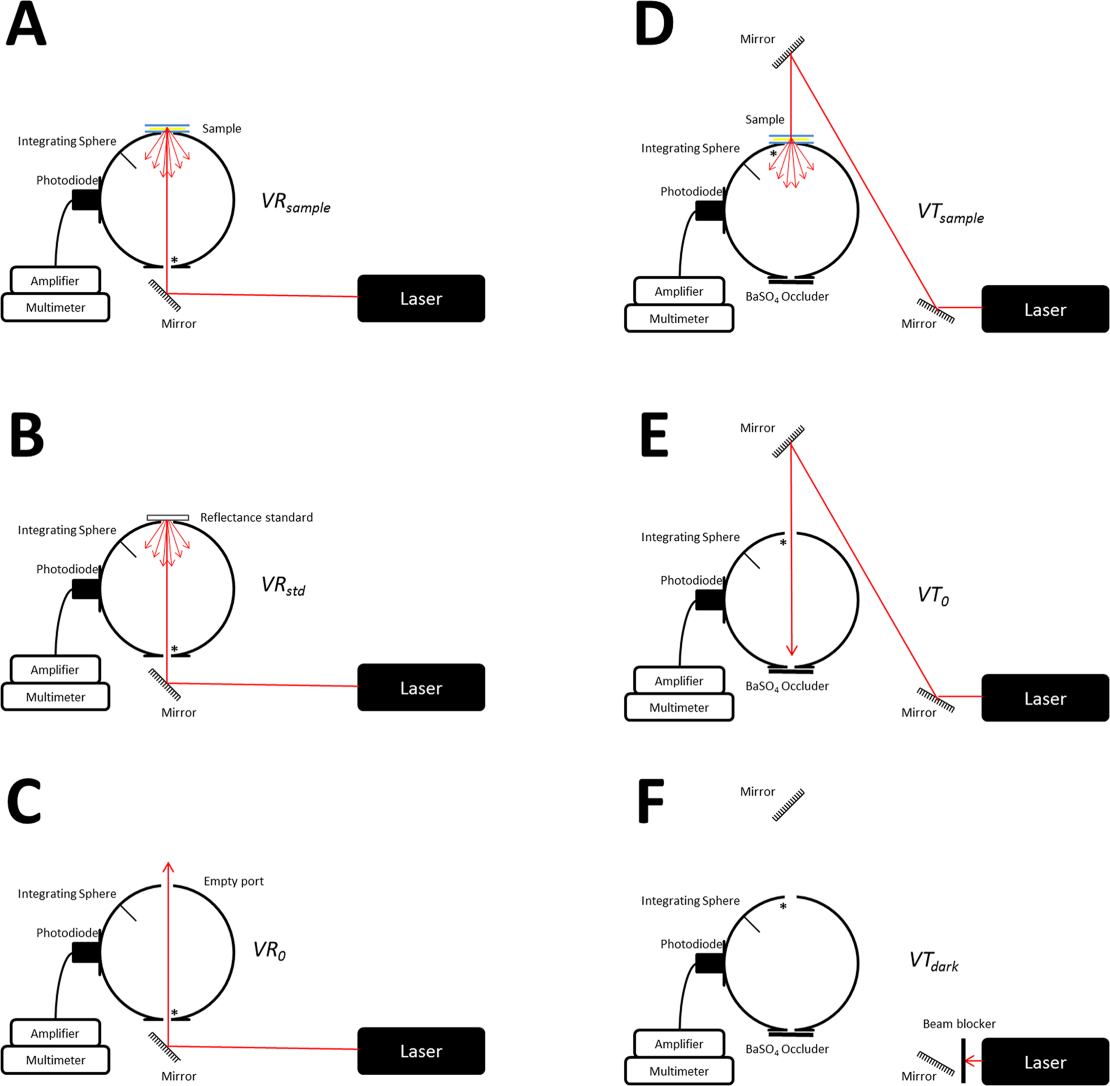
Schematic representation of the experimental setup for performing the IAD technique. A-C: Configuration for Reflection measurements. D-F: Configuration for Transmission measurements (* represents the entrance port for each measurement).

Fig 3 shows a schematic of the experimental setup for measuring the total diffuse reflectance and total diffuse transmittance. In order to prevent oversaturation of the detector (53–2754 Model, Coherent, USA) intensity of the incident laser beams using a neutral density filters. The signal from the detector was measured by a digital multimeter (34401A Model, Agilent Technologies, USA). Three reflection measurements were made on each sample, recording 50 data points each time, referenced to a 98% Optopolymer reflectance standard (OPST3-C, Optopolymer, Germany). Dark measurements where made with the sample port open. The PCCS was placed between glass plates to prevent dehydration and provide mechanical support whilst minimizing surface effects[27]. Reference sphere calibration measurements were also made and the reflectance of the sphere wall was determined[26], taking into account all the geometric properties of the sphere known (wall area, sample area and other aperture areas).

Total diffuse transmission measurements were made with the same integrating sphere using a mirror system to divert the laser beam in order to not move the sample after the reflection measurements, so that the reflection and transmission measurement could be made in the same spot of the sample. In this setup, only two ports were open, the 7mm-diameter sample port and the 10mm-diameter detector port with a baffle between ports. Again, three transmission measurements were made on each tissue sample (50 data recorded during each measurement). Measurements were referenced to 100% with the lasers illuminating the open port hole, and a dark measurement with an open port but with no illumination from the lasers.

The refractive index of plastic compressed collagen scaffolds was assumed to be 1.43, based on the results of Wang *et al*. for hydrated collagen films[28]. Changes in the refractive index over the wavelengths used in the present study were assumed negligible.

The total diffuse reflectance and transmittance measurements in terms of percentage were calculated using

$$R_{d\;sample}=r_{std}\frac{{VR}_{sample}-{VR}_{0}}{{VR}_{std}-{VR}_{0}}$$

and respectively,

$$T_{d\;sample}=\frac{{VT}_{sample}-{VT}_{dark}}{{VT}_{0}-{VT}_{dark}}$$

where *r*_*std*_ is the reflection of the reference standard, *VR*_*sample*_, *VR*_0_ and *VR*_*std*_ are the reflection measurements of the sample (Fig 3A), with the empty port (Fig 3C) and of the reference standard (Fig 3B) respectively; *VT*_*sample*_, *VT*_0_ and *VT*_*dark*_ are the transmission measurements of the sample(Fig 3D), with the empty port (Fig 3E) and with no light entering the sphere (Fig 3F), respectively.

Measurements of the reflectance and transmittance were repeated three times for each sample of PCCS. All the experimental data were, then, processed using the IAD method developed by Prahl *et al*. [26] in order to determine the optical properties of all samples for each wavelengths 457.9nm, 488nm, 514.5nm and 632.8nm. This method has been widely used in tissue optics for processing experimental data obtained using integrating spheres[29–32] since it offers the possibility to rapidly determine iterative solutions with the help of microcomputers.

### Statistical analysis

Normality (and homogeneity of variance) assumptions were satisfied so, parametric statistics were used. In order to analyze the relationship between the measured optical parameters (MT, MR, μ_a_, μ_s_’ and μ_t_’) and the parameters of TR and BI calculated from the pictures, the Pearson Product Moment Correlation were applied. This coefficient (ρ) shows the linear relationship between two of these variables. Applying the Bonferroni’s correction, a value of p≤0.005 was considered to be a statistically significant correlation.

## Results

The histology staining showed a well-developed structure of the PCCS. A fluid leaving surface was observed in all the PCCS analyzed. The average thickness value obtained from histological sections was 0.141±0.009mm. The histological structure of the amnion was similar between the different multilayering samples obtained. Therefore, all the samples used for its optical evaluation by IAD or photographic based method were histological suitable.

**Fig 4:**
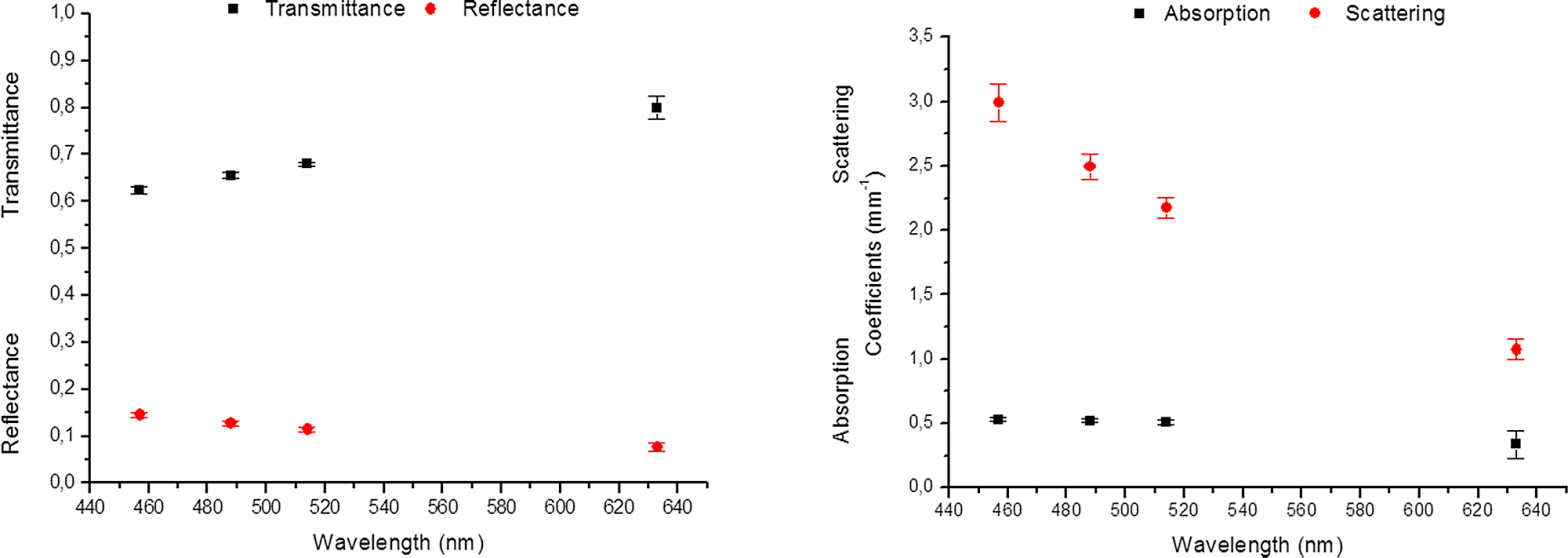
Spectral behavior of the optical properties of the PCCS obtained using the IAD method. A: The experimental reflectance and transmittance values, together with their standard error. B: The spectral behavior of the absorption and reduced scattering properties of the PCCS, as calculated using the IAD program.

Regarding the optical evaluation, Fig 4 shows the spectral behavior of the optical properties of the PCCS as it was obtained using the IAD method. The experimental reflectance and transmittance values are presented in Fig 4A. It can be seen that all PCCS samples presented transmittance values higher than 60%, values that increased with increasing wavelength. On the other hand, the reflectance values slightly decreased with increasing wavelength, displaying a maximum value of approximately 14.54%. These values of reflectance and transmittance were then used in the IAD program in order to determine the absorption (µ_a_) and reduced scattering (µ_s_’) coefficients of the PCCS. This program also requires additional parameters related to the sample studied, such as the refractive index, anisotropy factor (g) and sample thickness. The refractive index was assumed to be 1.43, according to Wang *et al*.[28] for hydrated collagen films, and the anisotropy factor was assumed to be similar to biological tissues, ranging from 0.8 to 0.99 ^[^ ^21^ ^],[^ ^33^ ^]^.Thus, in the present study we assumed a value of g of 0.9. The calculated absorption (µ_a_) and reduced scattering (µ_s_’) coefficients of the PCCS showed variation across the wavelengths studied (Fig 4B). The absorption coefficient presented an almost constant spectral behavior for the argon ion laser wavelengths, with values around 0.5mm^-1^. For the 633nm wavelength a decrease in the absorption properties of the PCCS was observed. Fig 4B also shows that the scattering coefficient values decrease with increasing wavelength, displaying the highest values for the lowest wavelengths.

The easily applicable photographic-optical evaluation method that we proposed in this study, in order to assess the optical behavior of the PCCS (S1 and S2 Appendices), indicated that these corneal substrates had a TR value of 80.3±2.8%, with a BI of 50.6±4.2% (Table 1).

**Table 1:** Transparency ratio and the blurriness index, applying the photographic-based method

	Petri dish	PCCS	1-layer AM	2-layer AM	3-layer AM	4-layer AM
**Transparency ratio**	1.000±0.000	0.803±0.029	0.951±0.012	0.859±0.016	0.774±0.014	0.699±0.026
**Blurriness index**	0.000±0.055	0.506±0.042	0.252±0.055	0.464±0.060	0.634±0.059	0.741±0.007

Transparency ratio and the blurriness index, together with their standard deviations of the PCCS, petri-dish, amnion (1 layer) and multi-folded amnion (2 to 4 layers), applying the photographic-based method.

The results of statistical analysis performed to compare the TR and BI obtained from the PBM with the optical parameters determined using the laser-based method (reflectance, transmittance, absorption and reduced scattering coefficients) are showed in Table 2.

**Table 2:** Results of statistical analysis performed to compare the TR and BI.

		Transparency	Blurriness
**MR**	*ρ*	-0.877	0.757
	*p-value*	<0.001	<0.001
**MT**	*ρ*	0.828	-0.632
	*p-value*	<0.001	<0.001
**μ**_**a**_	*ρ*	-0.528	0.312
	*p-value*	<0.001	0.001
**μ**_**s**_ **’**	*ρ*	-0.902	0.750
	*p-value*	<0.001	<0.001

Results of statistical analysis (Pearson’s Correlations -*ρ—*and p-value) performed to compare the TR and BI obtained from the PBM with the optical parameters determined using the IAD method. Reflectance (MR), transmittance (MT), absorption (μ_a_) and reduced scattering (μ_s_’) coefficients.

**Fig 5:**
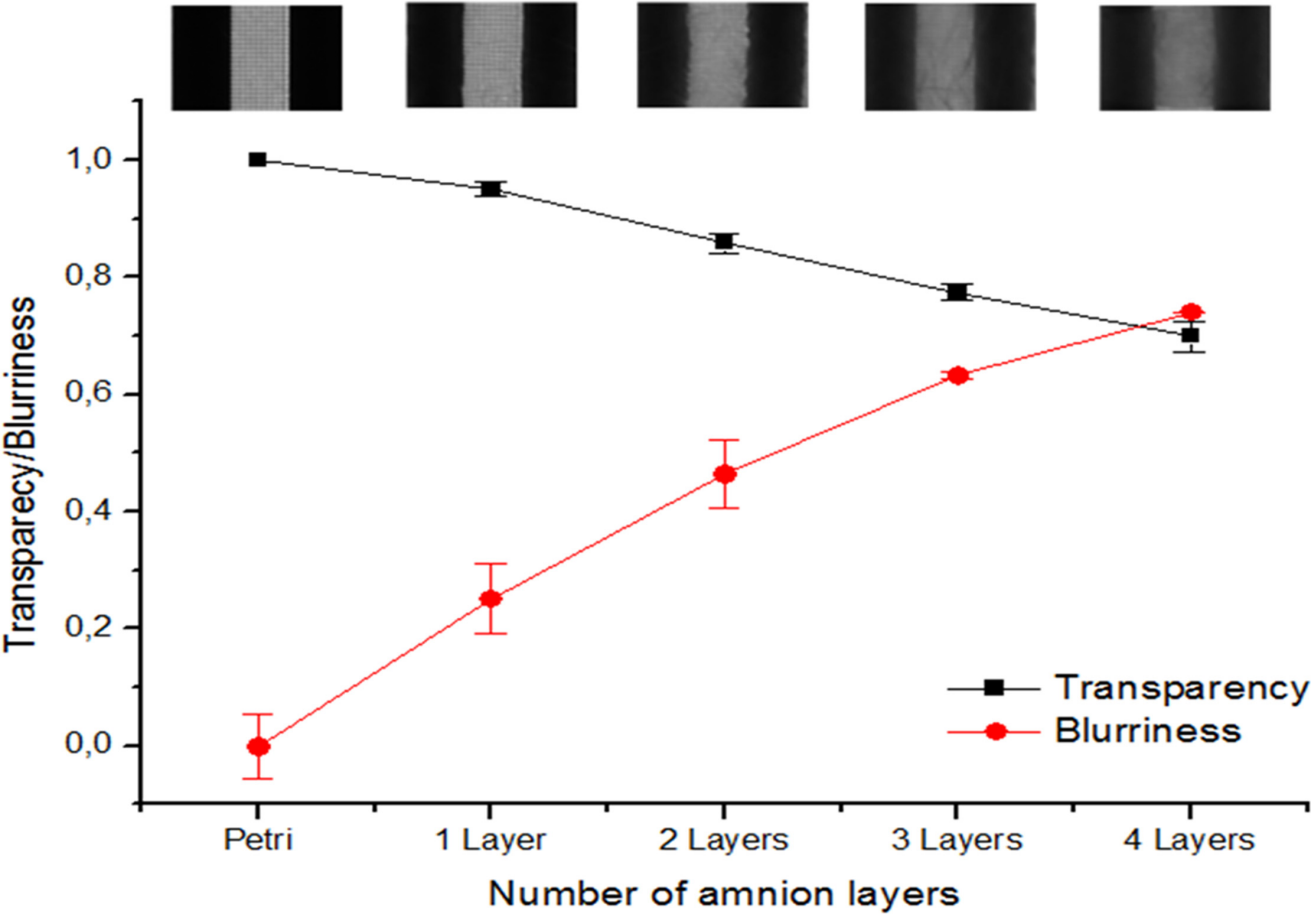
Transparency and blurriness using the photographic-based method. Transparency and blurriness of petri-dish, amnion (1 layer) and multi-folded amnion (2 to 4 layers), showing a representative image of each sample on top, using the photographic-based method.

Once the correlation between the two methods used in the present study was made using the PCCS, an evaluation of the AM optical properties was performed to evaluate this technique for ophthalmology applications (S1 and S2 Appendices). Fig 5 shows the BI and transparency measure for a series of multilayered AM constructs. There was a largely linear relationship between the blurring and the number of amnion layers, with more layers producing greater blurring. Obviously, transparency gave the reverse (inverse) relationships with increasing amnion layers, though the slope of this decrease was less pronounced (around half) of that for the BI, implying a less sensitive measure of function.

## Discussion

The new transparency measurement method used in this work give us an accurate value of transparency and blurriness based on a simple and cheap methodology. Optical evaluation of PCCS revealed that blurriness and transparency measure using a screen, a camera and ImageJ software analysis could be performed, obtaining accurate optical data. This was demonstrated by the high correlation statistically obtained between the new method proposed here and the gold standard, the IAD. However, the PBM slightly overestimates the PCCS transparency properties compare to the IAD transmittance values. This statistical analysis indicated a general, significant negative correlation, for all wavelengths, between the diffuse transmittance (IAD method determined) and the photographic-based BI method (ρ = -0.632). On the other hand, higher positive values of the Pearson’s coefficient were obtained for the correlations established between the BI and the diffuse reflectance (ρ = 0.757) and the reduced scattering coefficient (ρ = 0.750). These were statistically significantly different, though no significant correlation was found between the photographic blurring and the absorption coefficient (ρ = 0.312). All these data confirm that the major cause of transmittance loss is due to the predominant scattering (Fig 4), as it has been previously described in biological tissues [21]. On this regard, a sample with a higher absorption coefficient will be correlated with lower differences between MAX and MIN values but it will preserve the edges or the sharpness between black and white areas (or dark and light gray areas). However, BI highly depends on scattering coefficient. Thus, scattering modifies the values between the transition zone from MAX to MIN values, increasing the slope, and therefore, blurring the black white edge of the stripe pattern.

All the optical properties, determined using the IAD method, were also correlated with the transparency parameter obtained by the PBM. As expected, theses correlations were inversed relative to the blurring indices, with higher absolute Pearson’s coefficient values in all cases (ρ > -0.87). Once again, no significant correlation was found between the transparency and the absorption coefficient.

Particularly, the transmittance was especially high for the longer wavelengths of the visible light spectrum, in which both the absorption and the scattering tended to be lower. In addition, as mentioned before, the statistical analysis for the comparison of the two methods used in this study revealed that the main optical coefficient influencing the transparency and blurriness of all samples analyzed was the scattering coefficient. These data are consistent with previous studies demonstrating that the spectral transmittance function of the native human cornea is dominated by light-scattering processes.[5, 34]. Therefore, the new method that we propose could easily substitute the standard, quite expensive, IAD method as a first optical quality control of bioengineered materials and anterior grafts for ocular surface repair that must fulfill important transparency requirements.

The practical utility and applicability of this new method in Ophthalmology have been evaluated using one of the widest tissues used in reconstructive ocular surface surgery: the AM. The results presented in this work showed that the transparency values obtained using the PBM are similar to those displayed by Connon *et al*. (both higher than 80%), where the amnion transparency was spectrophotometrically quantified [35]. In addition, using also method based on spectrophotometric measures, Ventura *et al*. established a 60% threshold value for suitable tissues for human corneal transplantation[36]. Here, a new threshold value is proposed determined by the common value between transparency level and blurriness, which is the point where the transparency and blurriness lines intersect (Fig 5), which is 72%. Therefore, the AM employed in ocular surgery using the sandwich technique should be folded no more than 3 times if transparency is the major concern. In this case, we should take also into account the variability between AM from different areas of the placenta and the inter-donor differences, which includes thickness and transparency [37, 38]. On this regard, PBM could represent an ideal technique to characterize the transparency of the tissue prior to its implantation.

Complexity of the transparency measure is avoided with this new technique based on a camera and a laptop screen. Tablets and mobile phones, together with compact cameras could be used to apply this accurate, feasible and cheap method to tissues and biomaterials that must fulfill specific optical requirements. Thus, this innovative proposed method represents an easy-applied technique for evaluating transparency that can be performed on substrates used for LESC culture and for other ocular surface applications, in laboratories, operating rooms or GMP facilities.

## Supporting Information

S1 Appendix Gray scale intensities used for the transparency evaluation of petri-dish, PCCS, amnion (1 layer) and multi-folded amnion (2 to 4 layers) using the PBM. (PDF)

S2 Appendix Gray scale intensities used for the blurriness evaluation of petri-dish, PCCS, amnion (1 layer) and multi-folded amnion (2 to 4 layers) using the PBM. (PDF)
