# Correction: Photographic-Based Optical Evaluation of Tissues and Biomaterials Used for Corneal Surface Repair: A New Easy-Applied Method

**DOI:** 10.1371/journal.pone.0146205

**Published:** 2015-12-29

**Authors:** Miguel Gonzalez-Andrades, Juan de la Cruz Cardona, Ana Maria Ionescu, Charles A. Mosse, Robert A. Brown

The following information is missing from the Funding section: This study was supported by the research project JA TEP-1136 from Junta de Andalucía and MAT2013-43946R from the Spanish Ministry of Economy and Competitiveness.
