# Supplementary material for: Photographic-Based Optical Evaluation of Tissues and Biomaterials Used for Corneal Surface Repair: A New Easy-Applied Method
Source: PLoS One. 2015 Nov 13;10(11):e0142099. doi: 10.1371/journal.pone.0142099 (PMC4643926; doi:10.1371/journal.pone.0142099)
Supplement: S1 Appendix — (PDF) [file pone.0142099.s001.pdf]

PIXELS	PETRI A	PCCS A	PETRI B	PCCS B	PETRI C	PCCS C	PETRI AM	1LAYER-AM A	1LAYER-AM B	1LAYER-AM C	2LAYER-AM A	2LAYER-AM B	2LAYER-AM C	3LAYER-AM A	3LAYER-AM B	4LAYER-AM A	4LAYER-AM B
0,0000	28322,21	15335,71	21202,68	14702,12	15307,99	16316,05	5585,66	13403,31	7024,33	26644,28	17983,00	10164,19	14569,64	12111,00	16304,76	13582,80	13755,06
0,0033	34081,68	18052,62	27005,87	17195,42	22647,76	18065,59	6922,38	15691,16	7596,91	29191,45	18254,92	10892,36	15690,66	13457,75	17079,69	14290,12	14515,56
0,0067	38082,44	20899,23	34455,88	20109,73	28106,24	20148,95	12360,87	17948,08	8287,17	30124,87	18592,10	11910,78	16912,48	14945,24	18077,44	15067,02	15321,74
0,0100	39906,04	23548,40	38786,56	22596,37	32990,23	21816,19	19740,55	20774,38	9292,05	31220,24	18885,48	12919,51	18186,90	16838,05	19150,23	15789,38	16267,82
0,0133	39611,68	25697,64	41218,27	25181,57	37836,11	23493,29	27832,77	23399,36	10462,53	31387,25	19138,86	14246,60	19540,02	18652,66	20098,41	16439,71	17143,85
0,0167	37848,89	26938,79	41231,39	26722,87	40256,43	24668,11	32450,32	25675,10	11895,03	32373,24	19376,63	15632,26	20784,51	20658,95	20854,71	17200,66	18025,22
0,0200	35717,90	27207,00	38526,50	27783,40	40521,39	25010,39	33548,24	27529,53	13499,07	32645,29	19574,97	17398,64	21909,14	22103,77	21572,17	17930,08	18793,14
0,0233	34177,87	26818,43	36738,34	28173,14	38473,07	25243,17	34758,83	28616,43	15694,74	32641,87	19742,33	19101,18	22860,93	22894,81	22192,51	18505,82	19466,65
0,0267	34060,20	26236,25	32618,89	27688,13	34759,64	25454,05	33851,90	29592,55	18043,21	33907,04	19848,73	20775,93	23676,89	23657,68	22563,48	19052,32	20051,55
0,0300	35435,73	25928,60	31335,50	27460,98	34898,28	25349,36	34736,69	30100,17	20662,97	34307,34	19940,47	22223,99	24402,10	23830,47	23035,04	19356,02	20481,29
0,0333	37771,47	26113,49	33482,41	27074,74	34774,87	25741,98	35195,19	30840,53	23087,96	34682,46	20103,19	23184,00	24978,10	24356,88	23397,57	19666,03	20932,77
0,0367	40160,16	26839,42	35557,41	27346,64	35188,58	26185,48	34919,90	31586,98	25085,23	34069,13	20348,12	24072,55	25567,59	24678,06	23860,38	19949,39	21362,67
0,0400	41423,56	27916,93	40308,91	28130,42	38190,06	26472,92	37481,11	32029,65	26689,95	32989,36	20630,20	24694,45	26129,08	24927,71	24362,13	20195,49	21805,74
0,0433	41068,29	28967,06	42267,01	28826,12	40155,78	27237,04	38200,29	32734,88	27716,26	33211,16	20953,72	25339,88	26593,91	25618,04	24796,53	20475,41	22238,26
0,0467	39295,40	29625,89	42043,36	29823,74	41624,64	27416,26	38258,28	32918,02	28711,11	32980,09	21331,05	25900,72	27164,82	25796,88	25333,07	20685,76	22595,50
0,0500	36900,50	29663,54	40686,16	30146,32	42255,79	27195,32	37315,98	32834,11	29344,96	33518,96	21579,97	26376,97	27526,58	26050,39	25704,14	20934,77	22973,42
0,0533	35036,34	29105,54	36474,97	30042,50	39609,03	27073,05	35165,88	32749,24	29935,70	34405,91	21886,03	26767,28	27831,62	26187,97	25901,71	21129,13	23342,66
0,0567	34407,12	28337,83	34547,56	29750,80	36051,17	26751,71	35681,35	32479,16	30684,96	34665,70	22035,97	27125,36	28053,95	26117,88	26052,12	21311,30	23709,81
0,0600	35389,82	27760,88	32219,69	28905,26	35342,74	26638,13	34929,31	32731,00	31233,84	35723,88	22204,28	27537,17	28323,61	26356,17	26079,72	21507,09	23943,87
0,0633	37595,67	27741,20	31918,18	28717,75	34454,73	26919,46	34901,82	33021,55	31668,76	35569,23	22447,19	27866,16	28554,32	26464,83	26273,09	21694,74	24162,86
0,0667	39958,93	28284,48	36300,89	28701,51	35498,68	27079,08	36456,95	33266,98	31941,58	35011,11	22760,65	28130,91	28614,65	26721,84	26407,56	21931,34	24433,72
0,0700	41481,68	29208,74	39224,14	29160,33	38467,72	27305,93	36753,72	33831,27	32170,18	34579,98	23011,46	28374,78	28880,64	27175,54	26637,24	22149,81	24609,66
0,0733	41400,88	30190,39	42305,93	30160,22	39396,38	27989,43	38902,37	33961,04	32406,98	33722,11	23454,73	28591,93	29255,04	27392,53	26946,19	22240,84	24860,49
0,0767	39818,66	30813,12	42709,16	30670,64	41806,66	28091,95	38978,18	34149,32	32477,57	34149,04	24008,82	28887,06	29337,84	27763,39	27137,72	22296,61	25117,92
0,0800	37507,25	30812,62	40510,45	31208,51	42770,15	27983,12	37395,34	34091,69	32730,32	34331,57	24583,72	29282,78	29641,25	27720,45	27384,01	22403,96	25304,39
0,0833	35418,34	30225,14	38553,15	31181,30	40098,14	27884,68	36477,55	33661,73	32977,13	34507,19	25347,56	29520,26	29834,78	27613,25	27457,68	22613,61	25621,57
0,0867	34551,34	29369,97	33987,79	30560,53	37272,36	27482,89	34222,64	33656,60	33208,63	35535,82	26053,90	29762,95	29918,65	27717,64	27402,51	22811,95	25778,42
0,0900	35206,79	28703,66	32343,45	30111,83	35288,48	27482,29	34755,75	33575,63	33559,99	35623,53	26570,25	29825,68	30080,11	27486,08	27450,31	23107,48	25904,98
0,0933	37124,94	28499,44	33019,67	29333,54	33956,59	27599,06	35038,67	33782,56	33784,63	36127,27	26974,81	29684,12	30212,32	27669,07	27349,23	23427,23	26090,11
0,0967	39525,04	28864,66	34286,77	29295,10	35694,08	27633,03	34830,46	34376,07	34150,94	35569,57	27271,53	29749,80	30438,27	27823,06	27457,34	23716,03	26215,19
0,1000	41213,68	29650,77	39299,75	29699,75	38170,52	28052,13	37635,56	34501,42	34187,04	34518,18	27539,33	29793,32	30634,38	28022,66	27672,29	24022,01	26361,37
0,1033	41503,95	30487,14	41794,62	30185,61	38797,11	28582,66	38307,35	34797,70	34043,90	34595,34	27677,23	29895,49	30793,11	28685,85	27742,50	24252,34	26489,88
0,1067	40254,94	31071,11	42594,02	31116,26	41876,79	28649,44	38791,53	34784,01	33987,63	34369,02	27737,16	30109,41	31040,37	28872,29	28045,87	24307,84	26628,85
0,1100	38037,67	31178,73	41687,36	31498,69	42661,38	28681,45	38126,15	34340,79	33945,41	34758,56	27837,38	30194,33	31086,65	29144,27	28275,01	24426,52	26802,80
0,1133	35913,39	30804,79	38090,46	31621,79	40409,56	28465,11	35775,30	34388,02	34139,75	35078,18	27974,14	30372,56	31079,73	29007,00	28350,66	24500,79	26967,92
0,1167	34678,45	30147,54	36041,43	31401,69	38141,95	28052,55	35765,58	34257,81	34404,57	35236,30	28090,78	30534,05	31101,88	28776,63	28470,88	24580,79	27071,57
0,1200	34947,00	29552,68	32512,86	30577,32	34881,03	28185,33	34515,28	34456,81	34448,00	36399,50	28119,48	30528,41	31118,36	28801,72	28598,72	24643,56	27222,28
0,1233	36623,46	29301,61	31595,34	30208,85	33991,15	28094,37	34522,78	34875,80	34409,05	36389,55	28241,83	30611,68	31193,49	28603,57	28694,16	24841,60	27281,85
0,1267	38937,40	29490,73	35016,12	29811,28	36058,07	28070,65	35932,93	35005,96	34311,29	36047,04	28510,74	30725,33	31322,82	28765,24	28848,38	25059,08	27328,44
0,1300	40907,37	30067,23	37583,11	30041,00	37457,17	28624,51	36234,14	35384,48	34062,85	35171,20	28731,47	30852,06	31280,41	28884,13	28907,45	25140,82	27424,99
0,1333	41534,22	30808,52	41543,21	30792,83	38671,91	29057,96	38509,47	35331,07	33968,86	34069,22	28938,28	31080,87	31433,13	28901,39	29056,47	25251,73	27453,09
0,1367	40584,67	31362,09	42723,84	31250,86	41921,83	29281,60	38819,84	34898,70	33891,96	34431,91	29195,32	31069,68	31461,09	29353,88	29133,43	25301,87	27619,90
0,1400	38503,45	31536,58	41532,84	31959,42	42287,23	29362,63	37616,93	34720,77	33899,96	34346,51	29435,51	31163,39	31594,82	29372,60	29069,50	25208,57	27658,85
0,1433	36175,92	31290,51	39842,84	32020,43	40598,25	29043,69	36652,79	34253,64	34096,07	34633,95	29903,38	31180,07	31624,54	29431,12	29127,64	25226,97	27716,72
0,1467	34732,54	30770,69	35304,25	31634,43	38488,82	28706,41	34526,09	34368,06	34146,09	35284,15	30208,96	31018,63	31543,24	29307,80	29184,58	25326,33	27740,74
0,1500	34693,18	30233,23	33491,42	31219,09	34595,48	28794,54	35183,47	34716,83	34284,15	35339,77	30399,80	31042,36	31557,11	29011,84	29128,33	25498,72	27717,59
0,1533	36102,96	29937,77	32462,29	30383,21	34539,38	28548,82	34890,66	34756,70	34276,27	36289,07	30521,81	31091,60	31437,04	29078,79	29103,68	25722,87	27742,67
0,1567	38413,93	30012,78	32862,75	30210,45	36060,18	28581,13	34558,91	35370,93	34333,26	36146,64	30588,60	31107,55	31470,40	28913,31	29190,18	25739,36	27577,10
0,1600	40492,55	30420,19	37778,66	30388,64	36568,61	29017,80	37275,07	35473,30	34338,46	35610,50	30943,02	31465,42	31459,10	28970,32	29202,38	25766,95	27552,72
0,1633	41463,91	31003,80	40563,01	30763,63	38815,79	29229,07	37890,26	35201,57	34220,26	34999,45	31019,56	31463,55	31498,73	29308,84	29252,68	25737,87	27612,24
0,1667	40845,02	31527,59	42543,08	31630,27	41726,66	29635,71	38876,32	34997,48	34316,16	34140,13	31251,86	31364,22	31671,43	29305,47	29305,39	25608,88	27515,13
0,1700	38931,05	31718,44	42175,71	31969,43	42055,19	29672,90	38415,28	34400,42	34300,17	34521,21	31654,50	31294,28	31769,93	29617,01	29349,65	25649,32	27512,02
0,1733	36652,18	31554,07	39112,08	32250,08	41094,78	29233,96	35996,01	34298,91	34418,88	34367,82	31869,14	30951,64	31820,40	29528,57	29560,68	25691,91	27466,20
0,1767	34927,50	31147,76	37123,71	32044,12	38513,35	28958,06	35621,94	34380,58	34601,60	34503,50	32436,38	30897,13	31874,00	29322,11	29575,58	25724,51	27386,51
0,1800	34582,95	30686,69	33004,56	31273,84	34563,70	28784,88	34151,12	34566,43	34652,18	35378,25	32591,56	30884,74	31883,65	29254,22	29638,92	25853,90	27351,29
0,1833	35693,81	30368,54	31775,47	30808,23	35161,73	28486,19	34433,34	35118,48	34623,86	35598,34	32510,22	30996,57	31892,72	28998,33	29735,98	25812,61	27232,61
0,1867	37775,11	30327,92	33765,88	30194,30	35722,98	28690,85	35576,86	35247,17	34625,21	36123,59	32431,66	31304,43	32051,56	29089,73	29541,14	25703,80	27204,13
0,1900	39982,73	30585,37	35673,74	30240,46	35908,97	29021,03	35702,26	35484,14	34482,64	35907,13	32115,94	31364,82	32157,63	29078,00	29440,61	25625,58	27201,90
0,1933	41187,45	31017,12	40302,39	30838,59	38938,67	29161,27	38353,13	35342,26	34374,12	35096,67	32324,38	31445,17	32183,38	29042,14	29373,19	25421,44	27186,01
0,1967	40900,31	31425,24	42171,37	31316,68	41023,46	29767,22	38874,34	34680,83	34452,81	34892,82	32476,01	31350,46	32066,67	29431,68	29249,87	25301,71	27195,57
0,2000	39271,05	31647,39	41957,65	32095,05	41864,62	29806,66	38050,36	34477,48	34404,42	34380,56	32591,82	30964,99	31993,05	29376,38	29280,01	25259,61	27187,34
0,2033	37025,38	31573,02	40613,38	32218,70	41595,02	29421,08	36915,18	34271,20	34400,46	34718,60	33248,63	30799,21	31900,56	29289,27	29171,77	25252,19	27156,45
0,2067	35236,63	31278,04	36379,52	31985,81	38562,36	29159,31	34557,59	34480,49	34213,96	34790,01	33571,54	30592,38	31816,94	29075,97	29064,97	25199,80	27218,23
0,2100	34567,02	30933,23	34532,75	31612,39	35223,20	28710,65	35161,88	35018,07	33999,89	34813,87	33909,24	30597,02	31742,79	28747,99	29034,01	25195,94	27188,11
0,2133	35358,18	30620,56	32333,94	30662,08	35310,40	28505,57	34621,07	35191,23	33720,33	35897,26	33911,77	30808,66	31657,07	28801,53	28980,07	25085,88	27085,37
0,2167	37291,39	30490,89	31990,45	30383,14	34984,75	28700,91	34488,94	35735,71	33510,68	36064,02	33499,96	30917,39	31388,08	28614,12	29105,66	24996,09	27016,41
0,2200	39434,55	30636,87	36182,96	30251,97	35758,15	28777,13	36910,57	35612,35	33443,88	36140,38	33495,90	31108,09	31223,07	28527,53	29147,23	24833,92	27079,53
0,2233	40899,46	30928,46	38813,45	30437,23	38911,56	28996,84	37602,66	35031,07	33538,88	35765,99	33152,90	31011,76	31104,41	28737,05	29181,32	24735,45	27206,05
0,2267	40941,33	31254,20	41778,14	31170,82	40049,46	29704,28	39133,55	34655,42	33640,42	34823,37	33265,54	30728,47	30952,30	28724,64	29332,18	24543,03	27274,46
0,2300	39560,10	31452,39	42212,58	31519,67	41803,33	29683,75	38863,80	34051,95	33724,46	34981,35	33552,68	30592,01	30778,90	29018,03	29369,19	24504,91	27321,96
0,2333	37419,40	31436,71	40156,02	32014,63	42019,82	29441,19	36918,36	34202,31	33770,62	34851,42	33753,29	30308,62	30761,50	28931,55	29521,98	24513,20	27345,25
0,2367	35399,93	31231,79	38279,13	31897,37	38806,81	29158,12	36300,93	34550,60	33845,83	35037,53	34609,95	30333,32	30741,03	28615,63	29565,39	24389,30	27316,47
0,2400	34462,32	30864,79	33820,72	31191,34	36203,59	28537,94	34304,77	34626,49	33847,12	35838,26	34720,73	30515,82	30686,96	28372,29	29473,38	24304,69	27255,91
0,2433	34916,52	30544,43	32242,70	30673,19	35095,77	28487,65	34692,58	35346,25	33934,52	35969,69	34629,07	30520,78	30657,20	28073,16	29396,15	24194,72	27149,78
0,2467	36591,91	30367,71	32750,87	29873,59	34317,00	28467,95	35392,55	35411,13	34019,31	36410,26	34348,11	30849,70	30534,83	28095,97	29179,00	23970,25	27014,77
0,2500	38828,09	30377,55	33831,42	29771,22	36028,73	28300,01	35365,77	35383,74	34066,16	35892,09	33756,43	30823,22	30492,15	27908,88	29128,53	23806,10	26914,56
0,2533	40501,11	30563,63	38685,89	30069,37	38722,97	28693,77	38400,95	35079,36	34069,34	34883,50	33693,26	30503,94	30385,26	27931,51	29031,52	23610,00	26780,66
0,2567	40898,63	30795,67	41133,58	30417,07	39340,71	29306,99	39038,82	34186,32	34130,13	34865,52	33405,49	30371,68	30310,69	28177,38	29040,57	23486,83	26714,15
0,2600	39802,68	31004,50	41976,99	31167,99	41810,46	29338,54	38815,17	34000,13	34182,93	34261,34	33435,59	29929,38	30247,22	28307,40	29136,03	23445,11	26597,12
0,2633	37724,65	31039,14	41143,48	31326,48	41954,34	29207,56	37757,48	33748,86	34193,88	34431,55	33841,29	29852,84	30158,97	28490,79	29142,49	23323,33	26443,84
0,2667	35679,88	30858,91	37580,60	31320,38	39033,79	28788,09	35304,23	33778,35	34249,44	35173,45	33971,65	30027,64	30009,85	28245,68	29242,89	23193,86	26328,51
0,2700	34441,30	30537,48	35681,07	31006,39	37021,42	28116,18	35449,88	34165,73	34282,23	35311,48	34473,18	30070,12	29848,76	27878,26	29173,55	23042,86	26151,42
0,2733	34596,39	30147,76	32364,47	30083,62	34455,14	28122,57	34381,73	34035,02	34124,21	36319,11	34265,25	30311,32	29632,60	27643,32	29092,86	22924,55	26022,38
0,2767	36034,51	29887,90	31520,41	29588,64	33835,28	27794,70	34353,60	34301,27	33907,69	36004,44	33637,68	30191,98	29533,18	27146,29	28914,08	22767,31	25789,01
0,2800	38068,71	29771,21	34675,30	29145,06	36048,34	27638,84	36496,44	34205,02	33635,34	34931,51	33281,40	29841,68	29430,74	27041,22	28469,01	22566,81	25555,03
0,2833	39859,98	29793,12	36931,49	29201,88	37702,04	28185,92	37086,24	33513,79	33153,54	34327,42	32515,63	29667,56	29186,43	26891,39	28336,31	22445,09	25426,38
0,2867	40472,97	29955,41	40726,87	29804,61	38856,61	28540,45	39132,25	33166,00	33007,29	33312,04	32312,91	29487,11	29128,99	26821,93	28125,29	22361,57	25291,63
0,2900	39639,57	30034,81	41932,11	30082,64	41765,96	28689,93	39112,69	32552,09	33000,54	33436,31	32145,87	29421,92	28955,81	27095,31	27866,90	22227,71	25133,30
0,2933	37750,04	29946,20	40688,54	30675,01	41607,29	28563,08	37291,62	32526,19	32846,46	33070,35	31916,00	29213,07	28756,63	27004,22	27770,62	22227,23	24886,76
0,2967	35569,20	29670,08	38948,11	30613,43	39136,10	27828,58	36143,66	32319,24	32907,38	32359,72	32502,49	28963,89	28523,29	27097,26	27470,54	22076,86	24664,51
0,3000	34109,99	29159,35	34485,75	29961,79	37180,02	27138,50	33453,88	32041,79	32779,18	32650,49	32693,73	28834,74	28362,74	26802,78	27291,67	21816,03	24436,29
0,3033	33868,24	28530,50	32749,92	29261,27	33703,09	26924,68	33474,16	32748,45	32722,90	32263,97	33264,21	28690,36	28220,23	26396,36	27061,62	21717,18	24188,41
0,3067	34917,87	27910,39	31694,59	28112,55	33762,80	26330,07	32944,38	32972,19	32460,25	33123,29	33264,05	28560,60	28112,74	26207,28	26679,89	21611,88	23954,24
0,3100	36743,86	27475,33	31744,90	27698,03	35551,23	26166,59	31672,71	33396,29	32232,65	33709,48	32458,31	28437,23	27984,82	25773,06	26343,00	21458,62	23713,37
0,3133	38300,82	27228,93	36259,89	27591,05	35870,14	26481,56	34298,95	33224,37	32120,45	34488,18	31863,91	28611,66	28015,00	25674,84	25995,92	21355,79	23424,76
0,3167	38700,75	26974,09	38535,54	27686,19	37612,14	26380,36	35685,93	32081,46	31987,57	35133,16	29502,94	28520,66	27914,93	25578,43	25621,70	21326,84	23163,48
0,3200	37282,88	26632,21	39922,73	28356,86	39987,53	26600,18	38317,54	30961,53	32136,14	33895,05	26953,96	28323,98	27818,21	25311,56	25154,20	21294,61	22762,47
0,3233	34066,48	25958,32	38544,55	28222,16	39695,66	26096,68	40352,64	27582,69	32267,29	32297,33	23353,13	27741,38	27372,26	25560,47	24591,75	21055,60	22401,93
0,3267	29587,41	24877,99	34288,42	27924,62	37298,16	24672,81	41423,98	23942,53	32283,43	28781,66	19364,05	26214,51	26701,74	25376,43	23917,23	20820,75	21727,04
0,3300	24440,50	23397,49	30091,83	26784,21	32332,54	23181,66	42548,89	19588,39	32625,82	24667,87	15944,46	24659,92	25799,52	24921,97	23341,56	20531,81	20937,90
0,3333	19266,33	21512,63	22736,58	24756,70	26166,82	21354,73	37079,02	15257,46	32483,16	20321,72	13111,28	21978,45	24310,85	24098,22	22467,59	20153,79	20135,77
0,3367	14314,18	19456,34	16387,21	22315,34	22350,13	19011,10	28715,84	11975,13	32094,88	15987,43	11244,86	19344,81	22647,17	22702,29	21455,71	19877,45	19161,28
0,3400	10075,24	17330,87	10470,61	19264,54	15255,63	17097,10	18345,05	9520,97	31452,43	12639,66	9909,11	16749,63	20298,26	21513,87	20178,91	19514,65	18153,13
0,3433	6883,95	15260,30	5908,15	16736,26	8724,22	14776,04	9256,79	8173,83	30139,26	10088,28	9093,28	14242,49	18019,52	19762,76	18952,99	19006,19	17049,63
0,3467	4781,30	13365,89	4086,95	14361,97	5277,64	12762,62	4699,43	7328,39	28248,90	8693,08	8460,06	12502,95	15937,58	17906,28	17568,16	18567,30	16000,32
0,3500	3604,08	11763,13	3256,78	12455,54	3129,29	11428,20	3223,77	6793,73	25708,68	7796,50	7952,77	11287,31	14113,85	15954,25	16285,50	17824,60	15149,24
0,3533	2964,00	10518,17	2869,43	11048,00	2421,66	10085,76	2754,49	6341,33	23002,55	7271,34	7514,52	10551,13	12846,70	14205,00	15168,51	16769,32	14374,82
0,3567	2595,51	9628,83	2529,30	10033,39	2420,29	9163,30	2373,66	5958,47	19742,52	6845,78	7096,68	9919,53	11818,63	13124,02	14198,31	15621,90	13670,03
0,3600	2357,95	8961,34	2310,94	9351,40	2126,68	8413,86	2162,64	5559,94	16665,52	6488,95	6689,28	9465,62	11127,83	12197,61	13466,64	14409,84	13069,78
0,3633	2186,62	8439,72	2151,58	8784,74	1961,26	7899,21	2023,61	5170,19	13624,67	6130,01	6235,83	9060,77	10465,31	11484,45	12748,78	13317,87	12544,58
0,3667	2050,49	8008,27	2019,79	8314,41	1836,55	7551,30	1906,49	4792,63	11031,50	5735,95	5816,86	8623,45	10029,72	10919,06	12070,36	12322,00	11999,62
0,3700	1926,92	7656,03	1895,72	7960,79	1737,34	7275,99	1770,93	4435,16	9253,35	5335,58	5460,02	8191,92	9604,16	10472,74	11588,48	11673,50	11542,45
0,3733	1805,80	7353,26	1767,10	7632,71	1650,14	7072,38	1583,50	4102,36	8061,26	4967,44	5157,66	7765,49	9118,69	10039,47	11098,39	11122,08	11156,39
0,3767	1692,71	7070,24	1640,27	7339,20	1591,45	6874,57	1420,88	3841,60	7385,70	4655,70	4917,91	7348,77	8641,84	9641,90	10561,62	10619,32	10832,39
0,3800	1595,03	6798,59	1549,24	7072,87	1507,36	6700,92	1329,32	3617,86	6849,63	4392,39	4736,40	7001,01	8226,23	9217,79	10225,14	10233,71	10501,29
0,3833	1512,83	6545,53	1471,29	6833,00	1416,04	6557,06	1277,21	3426,64	6463,57	4158,79	4557,92	6686,31	7840,48	8800,55	9859,50	9885,31	10164,26
0,3867	1441,62	6322,51	1395,41	6589,33	1334,45	6392,62	1219,50	3276,74	6082,88	3980,48	4413,03	6419,04	7479,79	8450,45	9474,46	9550,82	9835,62
0,3900	1378,23	6121,65	1323,47	6359,93	1266,36	6210,39	1158,48	3143,35	5740,33	3819,89	4255,66	6187,20	7139,53	8101,81	9118,47	9313,67	9526,04
0,3933	1322,32	5938,37	1257,07	6171,62	1206,80	6046,79	1113,34	3005,54	5401,52	3681,25	4118,84	5978,21	6860,08	7765,27	8817,08	9044,54	9242,18
0,3967	1274,11	5770,46	1203,81	5985,86	1161,60	5886,81	1096,31	2874,59	5068,01	3567,81	3993,40	5785,97	6599,13	7461,88	8604,54	8753,54	9003,39
0,4000	1233,11	5616,20	1170,17	5807,03	1132,34	5749,13	1073,87	2764,54	4787,95	3464,37	3869,80	5589,26	6367,53	7204,81	8337,63	8457,93	8746,18
0,4033	1199,42	5475,17	1143,33	5653,14	1108,01	5619,20	1035,39	2673,73	4544,19	3350,92	3772,24	5390,61	6152,75	6976,44	8098,63	8244,25	8507,65
0,4067	1167,89	5347,98	1118,78	5501,07	1088,66	5503,99	1023,23	2607,98	4359,52	3242,34	3709,71	5226,83	5961,16	6741,36	7835,01	7979,60	8315,13
0,4100	1138,20	5232,86	1087,61	5375,11	1075,21	5405,90	1018,10	2530,67	4197,97	3154,95	3595,28	5074,03	5788,11	6549,36	7627,29	7766,23	8111,50
0,4133	1110,70	5123,12	1074,90	5257,39	1055,28	5304,95	999,68	2466,59	4055,43	3057,39	3507,94	4931,10	5606,12	6338,81	7460,24	7528,34	7925,84
0,4167	1085,37	5015,47	1048,04	5139,88	1021,11	5212,04	980,67	2386,28	3937,82	2990,05	3435,18	4831,63	5432,28	6141,66	7248,76	7332,35	7763,61
0,4200	1061,78	4920,92	1021,46	5028,08	997,64	5141,72	978,61	2315,10	3813,54	2926,01	3373,32	4694,01	5278,28	5958,75	7049,04	7156,24	7600,55
0,4233	1044,06	4833,37	1007,30	4930,92	994,01	5066,78	965,10	2266,62	3699,33	2868,04	3324,44	4557,60	5109,03	5804,34	6869,61	6939,69	7377,08
0,4267	1028,84	4760,89	993,84	4835,62	988,80	4982,70	953,29	2219,23	3603,97	2797,50	3289,94	4444,15	4953,19	5681,28	6690,79	6739,44	7198,51
0,4300	1015,01	4690,71	981,81	4740,70	970,49	4913,31	945,77	2176,10	3502,95	2758,25	3243,57	4326,80	4816,16	5548,93	6534,70	6616,50	7053,72
0,4333	1002,10	4623,04	967,91	4653,72	955,56	4862,95	950,56	2139,68	3413,77	2699,12	3199,25	4233,12	4715,44	5398,76	6373,96	6461,71	6921,53
0,4367	991,34	4558,62	958,41	4583,04	949,52	4812,63	927,46	2107,03	3325,33	2635,97	3164,30	4130,72	4578,83	5262,15	6242,21	6324,28	6732,92
0,4400	978,46	4499,51	948,46	4509,78	943,26	4751,65	914,91	2059,71	3240,58	2596,77	3125,88	4037,26	4414,07	5164,22	6084,10	6200,60	6589,32
0,4433	965,34	4434,88	944,15	4446,96	937,02	4689,96	910,69	2020,34	3142,51	2570,68	3077,16	3940,63	4323,33	5050,21	5940,85	6112,40	6464,42
0,4467	953,58	4373,93	936,35	4380,49	925,82	4636,95	904,73	1997,84	3051,94	2534,95	3042,98	3868,47	4232,05	4917,27	5817,96	6014,29	6348,08
0,4500	943,17	4327,74	930,25	4318,53	919,83	4589,51	905,32	1960,01	2943,67	2484,96	3011,89	3827,80	4150,31	4824,63	5676,92	5889,28	6227,45
0,4533	932,78	4287,15	918,94	4265,55	914,65	4541,58	881,54	1921,13	2857,67	2460,76	2990,13	3745,53	4069,21	4749,04	5563,87	5801,03	6102,03
0,4567	923,08	4239,74	909,53	4219,97	909,31	4496,21	880,20	1888,75	2801,17	2441,66	2988,62	3672,45	3988,62	4643,73	5470,66	5707,63	5990,71
0,4600	916,82	4196,33	898,78	4170,80	897,76	4455,52	878,04	1861,59	2725,63	2417,54	2978,38	3623,67	3904,71	4522,77	5389,13	5620,76	5868,79
0,4633	914,59	4159,96	893,56	4125,03	896,50	4428,60	864,18	1838,75	2647,53	2392,52	2954,70	3585,65	3839,96	4441,33	5316,03	5546,49	5788,26
0,4667	911,88	4126,20	893,88	4093,86	895,64	4396,71	873,96	1825,63	2592,56	2368,40	2954,52	3552,48	3802,71	4402,70	5203,02	5463,63	5739,13
0,4700	908,30	4097,39	890,50	4055,83	896,31	4364,20	875,86	1793,37	2522,38	2356,21	2974,96	3493,77	3773,02	4349,52	5101,60	5392,04	5641,88
0,4733	905,44	4070,66	900,19	4016,84	893,72	4337,79	871,14	1772,13	2466,27	2339,64	2979,52	3467,77	3754,13	4294,02	5035,75	5322,51	5575,45
0,4767	902,95	4041,73	899,81	3982,06	887,94	4313,52	867,95	1765,08	2411,50	2319,87	2981,75	3453,31	3715,43	4225,32	4968,72	5291,19	5526,39
0,4800	898,95	4022,99	876,67	3959,88	886,03	4286,81	856,28	1759,70	2359,15	2318,79	3013,32	3443,94	3677,93	4179,19	4918,02	5243,13	5489,85
0,4833	897,55	4012,69	868,32	3931,20	877,15	4261,79	859,35	1744,04	2328,64	2338,14	3035,73	3431,45	3668,24	4138,01	4885,62	5193,84	5439,29

0,4867	898,43	4001,21	882,23	3903,76	879,19	4248,22	853,01	1747,72	2296,39	2326,81	3038,30	3400,57	3651,95	4104,67	4808,90	5151,13	5392,46
0,4900	899,98	3996,88	879,38	3886,37	879,74	4246,55	855,57	1749,90	2256,00	2308,33	3055,78	3371,43	3633,77	4077,59	4781,69	5114,48	5369,58
0,4933	899,47	3996,93	873,62	3876,81	879,11	4243,47	861,82	1740,21	2237,82	2312,64	3078,11	3369,46	3616,52	4051,27	4769,40	5083,56	5356,73
0,4967	899,51	4000,70	872,47	3858,77	881,58	4232,20	851,56	1765,46	2212,35	2312,26	3090,38	3376,37	3632,11	4007,72	4765,17	5046,35	5334,96
0,5000	900,18	4003,76	877,70	3833,28	889,10	4224,70	857,23	1750,34	2198,94	2328,50	3111,80	3389,67	3636,14	4009,25	4761,19	5012,19	5298,72
0,5033	899,70	4012,10	878,99	3821,19	887,95	4229,50	872,92	1748,77	2189,80	2334,82	3147,68	3390,55	3642,66	3993,56	4772,59	5011,44	5280,86
0,5067	897,91	4012,74	875,03	3818,88	883,08	4231,59	866,89	1767,90	2184,81	2364,56	3182,80	3396,83	3643,23	4004,29	4787,08	4989,61	5294,50
0,5100	897,24	4007,94	874,30	3822,72	877,33	4240,07	851,47	1772,22	2203,62	2376,41	3229,40	3428,23	3666,93	3995,26	4777,92	4956,34	5300,66
0,5133	896,05	4007,44	880,49	3817,11	875,86	4248,64	850,97	1793,40	2214,34	2410,92	3271,32	3433,72	3691,57	3989,93	4790,96	4938,71	5299,41
0,5167	895,09	4001,61	875,57	3813,86	881,86	4256,53	848,94	1819,60	2204,07	2430,14	3296,23	3468,84	3717,12	3999,13	4823,37	4945,42	5314,85
0,5200	894,73	3992,19	870,30	3816,74	886,36	4265,54	851,73	1839,67	2205,96	2443,82	3327,78	3512,97	3767,21	3979,29	4840,79	4946,24	5306,76
0,5233	896,66	3986,97	870,13	3821,62	890,73	4275,75	843,23	1847,68	2243,66	2467,83	3364,76	3558,34	3804,00	3989,15	4866,87	4949,71	5319,71
0,5267	900,75	3985,55	875,45	3827,01	888,14	4297,80	854,78	1875,01	2273,46	2499,45	3375,34	3603,26	3821,32	4018,85	4891,87	4945,87	5336,67
0,5300	905,42	3989,76	884,44	3848,42	890,56	4335,16	870,17	1881,22	2288,82	2548,85	3412,09	3634,89	3871,34	4056,52	4938,89	4965,35	5374,93
0,5333	908,94	3998,88	885,87	3873,75	901,24	4362,71	873,21	1897,33	2304,09	2590,32	3441,85	3681,66	3934,87	4092,68	4996,96	4975,73	5398,18
0,5367	910,93	4014,53	890,59	3892,70	908,61	4375,41	870,43	1921,85	2349,50	2637,13	3488,97	3755,07	3994,18	4126,22	5020,45	5002,90	5449,31
0,5400	912,84	4037,32	889,89	3920,39	906,16	4408,77	878,17	1955,64	2382,34	2688,07	3556,27	3811,42	4052,57	4163,67	5069,72	5009,40	5472,31
0,5433	915,68	4064,23	894,53	3940,27	907,65	4443,28	895,72	1965,28	2399,77	2726,29	3617,77	3849,13	4110,48	4224,48	5173,81	5024,16	5510,34
0,5467	919,86	4095,03	897,37	3958,55	916,00	4479,21	899,92	1984,60	2411,63	2776,94	3666,00	3908,42	4187,66	4270,09	5229,25	5025,76	5571,60
0,5500	925,34	4126,15	901,59	3988,63	923,72	4522,19	912,25	2014,70	2449,05	2825,58	3709,19	3966,09	4252,64	4338,29	5269,04	5047,07	5609,78
0,5533	935,04	4162,33	902,46	4030,96	929,09	4561,20	928,68	2064,75	2498,47	2896,94	3800,28	4050,52	4347,23	4424,01	5334,09	5088,71	5672,58
0,5567	946,33	4195,83	913,68	4071,80	936,86	4605,06	950,79	2092,15	2541,44	2969,64	3893,80	4122,78	4433,19	4489,38	5421,82	5113,22	5742,52
0,5600	955,72	4236,07	923,76	4110,89	947,57	4657,10	941,93	2120,41	2577,14	3051,13	3963,67	4226,20	4520,74	4564,35	5519,57	5141,87	5820,91
0,5633	964,65	4287,73	927,91	4159,06	948,88	4713,32	940,97	2166,66	2639,30	3117,49	4053,99	4300,62	4583,06	4645,52	5641,32	5212,65	5905,44
0,5667	974,84	4349,57	929,29	4208,43	958,69	4776,39	950,16	2220,23	2690,47	3198,19	4153,26	4386,86	4670,88	4735,77	5743,60	5273,84	5991,35
0,5700	984,86	4418,28	940,34	4258,42	961,97	4843,29	975,38	2278,40	2748,26	3277,34	4238,88	4473,90	4751,20	4833,20	5832,80	5320,16	6087,90
0,5733	996,63	4495,22	953,76	4310,58	966,89	4903,26	978,59	2355,60	2798,41	3370,76	4326,87	4575,38	4798,46	4923,55	5927,01	5403,50	6206,12
0,5767	1012,76	4580,42	964,06	4370,00	980,41	4956,51	992,76	2429,43	2874,16	3467,11	4429,53	4687,04	4873,42	5028,72	6028,07	5489,13	6313,30
0,5800	1029,26	4670,96	969,63	4434,32	1000,83	5028,26	1000,13	2482,58	2927,32	3587,05	4590,69	4786,10	4977,51	5112,98	6146,55	5559,83	6448,80
0,5833	1046,31	4769,46	977,20	4502,72	1007,04	5101,38	1016,36	2578,18	3002,52	3696,00	4706,41	4872,58	5072,97	5245,83	6281,44	5651,93	6568,89
0,5867	1066,24	4875,02	1003,23	4583,64	1016,68	5183,87	1040,64	2686,98	3051,43	3802,71	4843,69	5016,28	5159,54	5344,06	6439,40	5743,27	6702,63
0,5900	1090,22	4983,21	1026,21	4664,87	1041,28	5277,31	1056,21	2779,70	3140,50	3925,14	4992,82	5149,74	5279,21	5454,68	6591,02	5844,74	6901,55
0,5933	1115,89	5086,50	1043,87	4744,38	1066,12	5366,79	1076,18	2896,51	3228,39	4045,17	5164,17	5297,80	5418,04	5592,58	6743,20	5931,03	7086,99
0,5967	1142,67	5197,85	1067,94	4830,61	1088,47	5462,81	1088,52	2996,97	3275,57	4182,72	5304,16	5440,80	5564,14	5690,36	6968,07	6036,63	7199,74
0,6000	1171,04	5311,11	1093,69	4928,42	1102,95	5580,23	1108,70	3120,13	3338,19	4311,45	5524,55	5573,62	5684,03	5813,45	7120,41	6158,32	7389,79
0,6033	1200,98	5431,05	1117,83	5035,67	1126,03	5704,40	1159,76	3257,70	3400,02	4429,57	5773,71	5716,55	5838,99	5974,12	7292,34	6276,77	7537,97
0,6067	1239,21	5557,43	1143,67	5143,85	1154,26	5825,50	1184,58	3387,32	3489,77	4561,39	6019,42	5871,38	5981,44	6121,88	7563,75	6377,68	7707,22
0,6100	1284,71	5682,79	1169,99	5268,78	1193,86	5962,65	1215,55	3497,19	3571,19	4664,54	6260,60	6076,80	6150,07	6265,31	7816,13	6475,54	7907,15
0,6133	1335,70	5821,52	1211,28	5397,57	1235,07	6096,33	1266,29	3615,20	3658,25	4725,13	6571,89	6280,27	6329,91	6410,48	8039,64	6622,50	8094,56
0,6167	1395,54	5985,58	1252,54	5534,75	1287,28	6252,04	1334,96	3695,39	3739,18	4728,13	6780,49	6430,56	6554,52	6613,12	8255,27	6765,92	8330,71
0,6200	1466,42	6149,56	1299,72	5686,18	1364,11	6409,23	1370,83	3757,43	3827,32	4703,73	7009,19	6544,73	6704,17	6815,77	8516,18	6947,47	8530,09
0,6233	1551,02	6333,63	1370,58	5873,80	1450,78	6583,34	1448,48	3885,93	3926,73	4735,68	7325,19	6717,01	6926,82	7023,08	8773,97	7135,50	8718,71
0,6267	1649,78	6532,23	1457,52	6068,00	1532,47	6800,15	1539,23	4066,30	4029,10	4775,55	7665,56	6908,33	7100,61	7220,63	9033,18	7327,60	8970,95
0,6300	1762,07	6725,27	1568,96	6276,82	1623,92	7059,82	1629,00	4285,42	4143,77	4878,21	8058,52	7150,35	7293,25	7511,49	9304,13	7485,94	9145,89
0,6333	1885,57	6920,24	1671,31	6480,53	1729,31	7313,13	1672,76	4677,00	4268,07	5036,81	8551,03	7474,94	7506,67	7722,54	9564,44	7700,28	9376,20
0,6367	2010,46	7135,43	1777,38	6688,03	1821,28	7514,19	1715,18	5176,37	4341,49	5313,67	9160,47	7922,58	7719,22	7972,24	9839,95	7933,56	9622,45
0,6400	2142,34	7376,15	1885,09	6910,08	1913,74	7704,49	1793,28	5944,23	4412,12	5561,43	10106,39	8510,88	7961,27	8181,41	10148,39	8157,82	9847,97
0,6433	2298,36	7670,70	2002,46	7171,81	2028,51	7944,56	1904,00	6848,06	4449,36	6196,51	11221,27	9388,92	8148,54	8387,88	10393,08	8402,64	10194,25
0,6467	2500,69	8042,34	2158,01	7425,62	2155,20	8228,54	2031,40	8139,06	4471,71	6794,74	12405,82	10465,10	8351,68	8652,64	10625,42	8670,39	10476,40
0,6500	2782,18	8538,34	2408,58	7797,70	2363,63	8582,07	2285,91	9700,58	4584,44	7701,47	14047,70	11610,38	8560,13	8905,48	10997,78	9000,73	10757,22
0,6533	3210,32	9163,87	2720,79	8249,03	2623,49	9055,50	2474,46	11618,03	4762,33	9061,12	15788,12	13294,20	8817,25	9140,89	11422,18	9333,42	11102,08
0,6567	3971,12	10019,52	3262,72	8808,28	3094,70	9594,83	2876,15	14440,47	4957,12	11296,75	17995,69	15012,39	9122,87	9418,40	11927,64	9677,42	11446,97
0,6600	5466,73	11149,48	4208,35	9504,07	3844,51	10281,88	3494,00	17476,76	5277,66	15470,30	20215,78	17264,94	9647,08	9767,52	12565,06	10033,83	11925,46
0,6633	8047,90	12535,09	6614,21	10574,19	6158,89	11271,40	4649,19	21458,01	5811,92	19735,42	22926,68	19393,40	10314,52	10131,13	13372,82	10485,39	12417,41
0,6667	11952,23	14221,21	9295,27	11769,76	10278,02	12744,26	5724,71	24825,39	6663,84	25249,86	24976,05	21749,82	11197,49	10747,68	14427,34	10879,08	12977,14
0,6700	17018,07	16216,85	14155,96	13459,65	15366,92	14701,58	7965,13	27717,20	7615,92	29052,38	26253,88	23811,49	12190,74	11572,30	15513,51	11318,92	13744,70
0,6733	22682,19	18453,04	20066,53	15370,53	21491,70	16802,19	13565,36	29912,18	8848,79	30806,50	27232,65	25145,55	13312,53	12681,48	16989,25	11773,17	14511,94
0,6767	28219,60	20816,52	25061,35	17675,43	27437,65	19067,22	19759,70	30620,63	10502,85	31687,94	27672,42	26190,57	14663,53	13914,90	18479,34	12405,11	15433,01
0,6800	32828,90	23134,88	31285,69	20255,71	30764,28	21386,22	28415,36	31137,41	12561,76	31141,61	28389,32	26693,98	16170,63	15323,88	20114,22	13125,79	16489,65
0,6833	36052,53	25142,51	35394,06	22585,85	35549,80	23465,97	33888,73	31066,45	15161,89	31853,59	28681,50	27301,07	17781,18	17169,63	21649,16	14121,82	17581,85
0,6867	37489,68	26732,85	38543,84	25163,57	38297,80	25491,92	36148,75	31479,06	17677,22	31861,15	29221,50	27479,75	19690,08	19080,82	22902,92	15254,22	18609,17
0,6900	37278,13	27705,55	39509,64	26748,16	38740,86	26601,15	36560,21	32090,18	20419,32	32208,71	30077,63	27860,88	21606,64	21114,42	23798,55	16601,07	19585,43
0,6933	36098,56	28041,48	37755,62	27949,62	37848,04	26947,94	34390,93	32571,95	22723,51	33819,07	30630,10	28572,46	23598,88	22578,32	24172,71	18114,76	20421,20
0,6967	34711,87	27972,91	36642,50	28460,94	34786,19	27181,53	34524,49	33566,12	24659,76	34489,00	31422,62	29131,47	25169,47	23338,95	24586,71	19349,45	21061,62
0,7000	33986,14	27761,84	33097,35	28155,49	33878,51	26910,41	33526,96	34039,32	26378,54	35511,58	31618,51	29977,45	26247,89	23918,29	24674,26	20427,45	21560,19
0,7033	34201,00	27697,75	32257,70	28056,69	35014,09	27201,57	33377,56	34324,90	27756,00	35317,09	31377,17	30280,42	26991,54	24071,23	24945,94	21044,49	21848,86
0,7067	35368,29	27899,77	32937,70	27618,57	35241,38	27379,41	36116,54	34290,11	28976,55	34266,30	31417,57	30417,53	27411,29	24463,33	25251,50	21599,28	22152,56
0,7100	37161,93	28397,48	33600,53	27752,75	36523,23	27444,84	37374,07	33616,66	30076,87	34057,63	31164,98	30553,47	27959,21	24634,09	25603,45	21994,70	22521,72
0,7133	38760,35	29096,05	37855,00	28300,14	39613,72	28096,58	39550,71	33678,74	31079,79	33395,71	31339,41	30274,24	28315,61	24974,49	26168,23	22157,14	22856,78
0,7167	39560,15	29766,16	39935,95	28913,13	40131,05	28905,63	39568,48	33587,99	31745,67	34038,01	31637,88	30403,18	28784,75	25753,95	26455,44	22547,68	23174,55
0,7200	39164,29	30219,68	40667,67	29950,95	40504,01	29238,26	37617,66	33777,33	32356,74	34313,07	31832,85	30391,70	29439,93	26269,61	26721,54	22784,39	23376,01
0,7233	37767,73	30313,13	39979,72	30364,71	39406,51	29447,46	36241,34	34425,93	32901,57	34436,09	32573,37	30527,43	29925,78	26960,22	26965,38	23090,30	23749,73
0,7267	36134,14	30101,38	36740,50	30524,04	35966,13	29242,35	33344,36	34614,29	33446,76	35672,57	32880,32	31096,97	30449,13	27324,62	26905,90	23491,82	24090,32
0,7300	34926,75	29762,78	35264,15	30459,58	35325,91	28785,17	33320,11	35185,93	34005,57	36005,83	33157,18	31304,19	30804,02	27342,40	27018,29	23687,55	24312,32
0,7333	34744,70	29523,41	32543,06	29757,13	34988,34	28893,14	34003,30	35381,02	34146,79	36633,63	33144,07	31634,22	30953,79	27454,46	27164,60	23964,23	24608,38
0,7367	35597,96	29541,67	31924,98	29489,14	34822,71	28715,62	34590,38	35020,18	34064,90	36393,18	32841,75	31677,11	31269,07	27218,71	27456,71	24037,71	24848,16
0,7400	37110,12	29810,40	34846,70	29208,45	37032,38	28735,53	38195,51	34678,59	34035,52	35470,95	32891,79	31329,88	31467,58	27333,64	27828,45	24057,55	25135,41
0,7433	38748,14	30254,40	36640,21	29412,53	39452,62	29336,69	39392,68	34033,99	33785,68	35206,60	32674,76	31183,93	31912,63	27533,78	28082,72	24187,96	25482,65
0,7467	39660,81	30726,75	39980,47	30182,86	40273,91	29788,76	39704,76	34151,38	34004,67	34353,06	32810,46	30813,57	32550,02	27720,63	28374,43	24229,73	25685,13
0,7500	39487,97	31024,20	41011,86	30665,94	41242,27	30207,00	38672,61	34438,04	34387,11	34768,89	33320,82	30898,46	32793,70	28329,27	28529,34	24361,14	25927,06
0,7533	38295,87	31076,07	39894,87	31339,92	39839,68	30429,54	35548,95	34567,10	34511,54	35295,77	33538,21	31272,23	33119,85	28599,48	28381,47	24525,89	26190,77
0,7567	36612,23	30898,23	38509,91	31441,11	36631,01	30100,75	34776,61	35346,74	34982,76	35567,96	34160,59	31418,72	33140,64	28791,10	28320,78	24723,12	26361,09
0,7600	35325,07	30563,21	34599,17	31058,78	35989,79	29754,73	33121,12	35517,54	35037,12	36924,99	34183,24	32064,95	33086,96	28772,19	28209,77	24977,55	26479,69
0,7633	34871,08	30289,08	33584,97	30819,16	34645,51	29704,01	33151,98	35763,50	34998,16	37058,82	33935,27	32221,99	33206,90	28522,08	28157,03	25064,18	26486,00
0,7667	35457,46	30210,87	33092,38	30191,26	35042,86	29373,44	35622,12	35713,32	34861,51	36868,66	33751,23	32111,22	33108,57	28522,74	28264,93	25103,88	26540,51
0,7700	36887,29	30381,16	33212,22	30087,40	37482,80	29491,87	37061,54	35145,84	34341,90	36287,80	33390,88	31935,16	33286,69	28330,73	28287,60	25146,30	26664,40
0,7733	38476,43	30770,14	37278,71	30247,12	38633,77	29914,13	39751,85	34962,56	34252,04	35041,09	33484,68	31490,88	33539,13	28371,13	28427,45	25115,41	26762,07
0,7767	39594,52	31196,18	39270,03	30635,52	40143,94	30098,70	40056,69	34466,29	34321,63	35108,49	33481,18	31481,50	33693,19	28758,99	28600,67	25229,33	26848,09
0,7800	39647,79	31527,07	40820,12	31415,00	41352,25	30679,71	38504,95	34443,68	34535,46	34785,47	33441,99	31403,98	34049,75	28968,41	28743,72	25189,42	27015,13
0,7833	38624,01	31624,55	40633,50	31649,15	39665,78	30918,71	37116,47	34894,57	35182,04	35033,54	33976,38	31366,75	34055,67	29563,61	28837,68	25275,81	27178,70
0,7867	37054,01	31427,31	37993,91	31859,86	37108,46	30640,02	34006,59	34960,48	35374,34	36137,70	34116,79	31958,76	34059,98	29646,58	28918,87	25399,54	27215,08
0,7900	35600,82	31088,67	36496,14	31823,52	35870,41	30388,00	33861,94	35768,66	35642,17	36409,85	34323,26	32187,67	34043,82	29588,09	29183,79	25539,73	27332,87
0,7933	35006,41	30733,97	33195,14	31247,55	34184,14	30106,71	33914,69	36038,73	35511,57	37315,33	34192,93	32485,16	33747,73	29610,65	29269,43	25729,40	27438,92
0,7967	35414,05	30552,12	32484,89	30930,78	35413,29	29784,38	34265,29	35838,77	35061,74	37237,83	33799,43	32506,28	33746,30	29217,09	29525,80	25771,30	27474,65
0,8000	36676,78	30667,05	34278,95	30515,22	37473,23	29942,77	37680,95	35557,68	34821,38	36503,71	33722,13	32127,04	33738,06	29163,91	29709,12	25698,42	27671,20
0,8033	38361,02	30978,72	35432,80	30556,82	37793,26	30168,84	39061,81	34811,68	34462,77	36262,42	33431,46	32060,43	33820,07	29121,39	29855,52	25747,25	27770,45
0,8067	39601,66	31380,51	39197,57	31029,48	40205,87	30289,67	40084,72	34740,42	34551,52	35392,67	33444,57	31764,77	34078,63	29132,98	30222,74	25687,09	27829,64
0,8100	39890,73	31721,77	40727,31	31383,89	41303,47	30951,20	39497,77	34799,09	34847,09	35582,47	33781,19	31807,93	34196,68	29683,77	30345,02	25724,19	28039,10
0,8133	39046,79	31842,64	40464,86	32048,92	39868,02	31081,45	36872,68	34809,14	34991,36	35965,50	33975,86	32092,49	34347,79	29977,31	30307,25	25751,72	28117,92
0,8167	37437,45	31730,10	39333,70	32211,46	38026,53	30899,35	36047,69	35659,18	35698,06	36151,48	34530,23	32174,03	34288,82	30073,42	30286,32	25924,64	28246,30
0,8200	35912,61	31440,13	35647,95	32079,09	35767,10	30719,46	33666,65	35896,59	35781,55	37076,23	34591,59	32649,77	34104,40	30010,95	30204,98	26159,78	28402,82
0,8233	35036,98	31130,09	34425,73	31838,27	34225,09	30221,04	33371,82	36086,54	35937,40	37030,32	34468,66	32812,54	33983,86	29626,45	30188,13	26142,65	28395,13
0,8267	35219,28	30957,05	32784,71	31138,66	35876,50	29953,61	35214,07	35846,23	35875,14	37242,72	34216,06	32625,19	33806,82	29627,25	30070,14	26297,39	28436,95
0,8300	36373,91	30987,14	32545,30	30911,39	37093,72	30008,44	36424,44	35133,38	35478,23	37078,72	33732,89	32252,54	33774,82	29318,35	30062,67	26364,40	28430,92
0,8333	37970,32	31233,79	36198,08	30823,70	37274,89	29996,44	39615,18	34789,48	35471,80	36132,56	33679,94	31704,03	33832,24	29261,00	30176,93	26215,48	28442,57
0,8367	39407,59	31621,94	38129,74	31043,07	40222,55	30259,38	40502,93	34219,34	35223,04	35991,88	33483,23	31604,53	33862,19	29684,94	30208,25	26302,06	28509,30
0,8400	39905,73	31962,17	40480,78	31717,98	40973,75	30950,25	39834,12	34258,13	35457,98	35445,71	33470,73	31379,72	34142,74	29894,09	30383,91	26197,13	28553,55
0,8433	39267,70	32069,48	40786,29	32012,31	40119,86	31061,41	38682,65	34726,04	36025,04	35674,09	34098,46	31352,62	34287,67	30260,45	30468,45	26215,23	28565,30
0,8467	37821,63	31899,68	38782,66	32376,68	38682,84	31032,95	35424,85	34857,36	36250,02	36483,96	34280,29	31754,29	34439,42	30309,99	30419,93	26389,83	28521,61
0,8500	36187,89	31520,30	37425,29	32340,67	35612,56	30859,16	34903,17	35538,79	36507,82	36858,10	34536,21	31916,80	34405,34	30163,63	30470,60	26451,83	28482,58
0,8533	35203,26	31081,71	33934,12	31763,38	34741,64	30295,31	33954,23	35776,50	36317,65	37936,52	34374,01	32276,26	34370,15	30027,79	30317,92	26614,97	28396,70
0,8567	35164,22	30790,84	33175,75	31437,17	35966,90	30177,94	33959,98	35581,87	35618,60	37865,23	33919,45	32294,61	34374,30	29592,22	30239,89	26561,40	28300,05
0,8600	36088,83	30780,49	33857,38	30899,75	36288,11	30057,15	37286,96	35346,45	35317,10	37307,02	33830,66	32081,62	34350,70	29510,19	30334,63	26503,92	28254,13
0,8633	37656,90	31059,18	34328,75	30855,21	37314,84	29927,55	38690,50	34580,14	34863,63	36830,09	33334,64	31928,00	34241,82	29461,64	30444,37	26455,77	28157,73
0,8667	39109,12	31512,82	38231,10	31140,84	40286,71	30290,08	40379,28	34496,40	34871,17	35464,13	33394,62	31388,03	34268,20	29341,75	30633,02	26239,77	28078,18
0,8700	39862,46	31912,48	40110,52	31482,27	40616,31	30772,08	40096,48	34291,35	35286,68	35388,09	33391,82	31322,98	34180,65	29848,38	30621,29	26249,11	28042,67
0,8733	39514,79	32089,05	40648,59	32263,93	40337,01	30933,31	37919,04	34434,18	35463,96	35279,67	33384,55	31279,16	33914,48	30143,22	30611,74	26252,52	27946,33
0,8767	38240,04	31947,07	40003,15	32481,97	39059,54	31063,75	36939,77	35158,01	36051,43	35349,69	34036,71	31384,61	33778,88	30463,58	30550,98	26277,28	27876,19
0,8800	36703,67	31552,10	36808,30	32467,88	35716,13	30793,70	33976,88	35405,13	36051,63	36667,77	34070,65	31910,79	33560,52	30359,40	30330,66	26371,79	27739,08
0,8833	35493,46	31128,06	35552,89	32169,06	35431,57	30251,45	33510,61	35898,46	35785,66	36948,39	34017,18	31995,98	33474,77	29931,73	30266,18	26313,02	27613,39
0,8867	35194,53	30837,73	33200,68	31380,28	35649,77	30159,44	34792,29	35856,69	35533,92	37373,05	33788,21	32209,53	33626,68	29795,90	30144,22	26391,35	27599,34
0,8900	35878,25	30793,65	32627,35	31083,72	35515,64	29831,79	35635,91	35223,63	34909,82	36888,83	33348,65	32088,95	33661,84	29395,17	30129,27	26295,43	27537,99
0,8933	37235,64	31032,58	35385,87	30802,45	37480,50	29678,99	39100,46	34858,07	34806,49	35642,74	33438,95	31516,72	33739,92	29309,08	30330,15	26074,20	27595,28
0,8967	38786,38	31427,98	36816,40	30932,13	39829,71	30064,39	40167,85	34335,98	34733,38	35297,02	33171,82	31285,10	33665,56	29611,42	30293,07	25947,29	27597,77
0,9000	39708,63	31802,89	39717,76	31510,01	40356,23	30318,89	39978,49	34314,65	34748,94	34463,42	33103,74	30866,77	33452,14	29673,90	30400,94	25655,73	27660,34
0,9033	39618,82	31986,13	40735,61	31835,61	40590,25	30592,52	38947,23	34406,14	35274,02	34491,75	33490,33	30822,18	33421,07	30166,78	30409,23	25636,41	27602,70
0,9067	38563,72	31878,79	39603,52	32304,64	38933,43	30752,64	35643,09	34461,62	35342,43	35208,76	33654,49	31278,00	33217,60	30137,20	30156,66	25671,66	27548,45
0,9100	36994,41	31496,96	38371,35	32287,90	35804,75	30392,56	34800,82	35136,63	35423,37	35513,34	34070,12	31324,38	33180,27	29923,28	30033,13	25659,64	27522,26
0,9133	35725,60	31009,59	34781,14	31784,51	35825,02	29934,56	33358,80	35357,87	35282,93	36855,86	33993,54	31832,56	33043,17	29646,63	29620,17	25740,91	27493,97
0,9167	35194,21	30655,46	33780,34	31385,55	35170,06	29747,14	33241,86	35573,19	34875,54	37009,43	33770,07	31913,61	32896,88	29021,11	29395,28	25613,04	27333,89
0,9200	35645,72	30572,99	33352,43	30672,67	35333,30	29306,67	36329,64	35391,56	34690,77	36390,74	33641,17	31645,81	32971,64	28799,34	29362,90	25345,96	27173,08
0,9233	36936,90	30752,77	33372,14	30564,16	37796,40	29258,41	37922,90	34670,46	34324,94	35638,17	32990,31	31291,99	33125,66	28558,07	29314,92	25290,10	27095,28
0,9267	38447,75	31111,12	37333,22	30643,65	39105,96	29553,77	40166,51	34370,68	34330,71	34292,82	32939,99	30710,03	33243,95	28405,77	29479,41	25082,05	27103,92
0,9300	39577,43	31483,97	39300,55	30893,29	40261,32	29633,61	40317,09	33753,39	34545,93	34405,66	32867,96	30511,40	33136,70	28890,89	29408,23	25035,13	27026,76
0,9333	39700,74	31631,85	40686,42	31649,67	40955,92	30004,94	38421,34	33758,00	34700,57	34408,12	32872,31	30330,08	32944,48	29042,47	29265,85	24987,31	26891,85
0,9367	38752,16	31470,07	40493,88	31789,72	38999,61	30079,75	37115,52	34084,63	35259,29	34698,23	33599,17	30272,74	32724,86	29120,86	29197,66	24975,98	26750,50
0,9400	37211,10	31021,38	37838,43	31813,04	36413,46	29728,52	33834,44	34080,21	35270,04	36218,89	33659,23	30670,07	32320,60	28996,40	28818,53	25016,53	26569,81
0,9433	35712,16	30447,52	36463,96	31638,37	35770,29	29392,27	33371,68	34704,52	35347,81	36325,44	33791,90	30840,33	32184,47	28499,22	28615,23	24921,29	26414,48
0,9467	35009,79	29971,60	33430,77	30874,95	34667,18	28983,11	34062,86	34835,81	35227,42	36427,31	33478,50	31037,48	32148,84	28374,31	28421,09	24848,13	26276,08
0,9500	35283,01	29764,76	32656,27	30429,17	35651,89	28507,97	34589,15	34567,55	34613,74	35850,93	32730,95	30875,47	32079,68	27827,77	28224,00	24641,96	26015,10
0,9533	36385,19	29898,91	34464,39	29931,05	37793,28	28436,02	38305,82	34146,75	34467,15	34425,82	32397,64	30294,68	32000,38	27517,15	28350,70	24257,29	25922,64
0,9567	37945,51	30230,33	35486,56	29854,66	38183,65	28423,42	39925,52	33227,92	34033,92	34049,72	31694,28	29897,33	31873,86	27829,85	28395,20	24021,85	25791,18
0,9600	39154,94	30595,63	38973,88	30239,61	40107,22	28381,33	40601,49	32743,29	34056,38	33239,14	31412,48	29241,03	31889,36	28057,03	28421,90	23676,63	25514,80
0,9633	39495,33	30806,57	40371,25	30411,60	40684,34	28881,27	39955,77	32264,28	34482,37	33024,23	31430,79	29040,78	31845,34	28656,35	28286,53	23491,12	25445,18
0,9667	38750,11	30693,30	39924,25	30840,27	38738,40	28925,39	36998,39	32034,60	34601,70	33674,07	31266,13	29217,25	31878,20	28768,97	27874,95	23387,23	25378,29
0,9700	37231,63	30208,73	38852,94	30802,04	36882,18	28648,54	35805,58	32373,97	35104,11	33528,58	31813,63	29212,57	31646,13	28422,73	27543,28	23187,11	25275,61
0,9733	35699,67	29479,18	35250,97	30298,13	35174,55	28260,50	32919,13	32298,84	34961,88	34635,04	31695,66	29884,22	31329,13	28040,53	26918,89	22974,62	25055,96

0,9767	34682,67	28786,79	34163,46	29728,07	33978,11	27610,89	31773,94	32775,36	34492,21	34986,83	31392,18	29871,78	31095,49	27196,13	26358,77	22606,09	24888,89
0,9800	34618,38	28294,29	32649,50	28720,48	35505,74	27105,43	33574,66	32838,23	34120,75	34965,07	30633,58	29163,73	30927,60	26700,83	25989,62	22217,61	24638,23
0,9833	35406,75	28053,79	32044,85	28222,55	36728,29	26765,31	34417,91	32231,82	33367,96	34718,25	29171,53	28185,69	30743,97	26261,07	25729,12	21864,86	24353,18
0,9867	36510,30	28033,33	35229,78	27886,38	36344,13	26365,43	37711,30	31253,45	33222,17	32403,34	27737,35	25812,82	30668,09	26081,16	25783,29	21394,92	23961,71
0,9900	37292,48	27997,31	36181,43	27761,47	38297,50	26237,72	39491,07	28858,24	33292,54	29776,25	24891,78	23559,49	30474,85	26636,22	25391,91	20979,63	23692,82
0,9933	36870,18	27674,30	37569,23	28177,21	37925,75	26410,00	40549,07	26295,87	33202,45	25374,82	21963,88	20573,03	30383,32	26678,74	25040,12	20594,15	23420,45
0,9967	34915,22	26837,54	36511,75	27953,10	36106,87	25978,17	40629,18	22611,64	33725,04	20616,19	18725,32	17551,98	29944,39	26763,35	24574,30	20229,29	22966,56
1,0000	31662,89	25392,33	32669,49	27643,80	32479,51	25340,51	36399,85	18942,42	33738,16	16058,96	15675,51	14914,74	29062,39	26348,26	23614,38	20004,41	22428,19
1,0033	27677,98	23399,86	29077,09	26571,59	27099,59	24216,57	31200,92	15528,45	33987,31	12026,44	13507,13	12690,64	27911,41	25094,14	22477,45	19467,21	21909,16
1,0067	23464,97	21067,78	23301,70	24627,02	23319,50	22400,03	21856,67	12515,22	33937,77	9629,69	11780,59	11330,76	26048,69	23707,11	20763,02	18979,92	21252,85
1,0100	19076,27	18685,46	18880,98	22456,18	18778,73	20846,93	12267,72	10637,18	33399,32	8148,79	10663,22	10335,94	24164,72	21238,71	19184,35	18300,79	20403,75
1,0133	14739,38	16375,43	13036,20	19774,79	12013,13	18815,02	6060,81	9262,99	32794,77	7485,36	9833,73	9685,03	21790,18	18862,90	17457,07	17359,57	19683,44
1,0167	10836,42	14261,08	8248,12	17481,50	7156,57	16705,02	3280,87	8475,65	31013,41	6938,28	9275,63	9182,17	19478,52	16432,88	15929,76	16142,35	18789,53
1,0200	7760,05	12491,93	5804,19	15173,02	4750,82	14889,94	3014,56	7866,15	28979,72	6541,17	8746,38	8789,38	17357,02	14260,08	14739,71	14788,95	17799,94
1,0233	5632,14	11119,50	3926,19	13309,18	2653,53	13229,48	2556,84	7423,50	25870,58	6210,93	8242,03	8401,54	15345,90	13003,69	13689,58	13780,83	16800,69
1,0267	4259,48	10111,19	3324,42	11758,52	2513,88	11847,00	2299,92	6994,04	22370,07	5867,28	7723,06	8007,82	13620,54	12021,61	12933,96	12768,70	16016,66
1,0300	3431,73	9357,14	2760,13	10573,37	2199,63	10882,68	2147,90	6515,03	18522,48	5456,38	7254,81	7630,91	12125,57	11255,25	12297,13	11944,41	15120,97
1,0333	2904,36	8754,11	2412,18	9729,61	2000,11	10052,62	2015,46	6081,17	14974,08	5041,73	6791,17	7290,04	11117,76	10616,80	11703,58	11199,14	14389,18
1,0367	2549,00	8294,10	2164,37	9054,88	1837,73	9447,63	1904,83	5632,93	12227,81	4700,46	6393,77	6962,21	10301,35	10172,27	11155,34	10671,07	13736,82
1,0400	2296,83	7940,42	2004,90	8581,43	1721,29	8904,80	1722,47	5214,27	9915,42	4417,25	6005,46	6649,63	9717,82	9707,51	10765,40	10295,45	13165,50
1,0433	2109,64	7638,79	1885,54	8200,13	1624,89	8531,09	1513,72	4877,75	8540,93	4160,97	5667,81	6390,05	9216,47	9258,35	10341,78	9921,25	12630,61
1,0467	1959,70	7358,92	1766,15	7842,53	1552,63	8192,96	1390,39	4573,31	7669,69	3957,71	5344,49	6174,71	8748,05	8846,45	9944,21	9619,64	12122,00
1,0500	1827,07	7096,43	1644,69	7535,91	1495,23	7890,15	1303,05	4294,66	7176,95	3768,79	5087,67	5972,58	8318,53	8450,19	9540,29	9398,82	11673,49
1,0533	1702,00	6836,09	1526,43	7238,33	1422,46	7632,07	1249,94	4048,95	6736,75	3657,87	4869,87	5796,51	7881,83	8027,19	9265,57	9138,83	11282,38
1,0567	1587,26	6595,72	1417,10	6966,91	1340,19	7412,48	1206,49	3819,43	6361,59	3546,28	4710,27	5626,38	7509,24	7662,05	8953,67	8870,69	10892,34
1,0600	1487,08	6373,74	1337,09	6695,56	1269,39	7194,55	1151,97	3645,95	5984,31	3423,65	4553,48	5459,09	7154,98	7390,41	8654,82	8674,94	10499,63
1,0633	1402,65	6173,05	1273,63	6463,36	1219,99	6970,69	1109,69	3489,69	5606,47	3319,57	4389,49	5294,55	6826,42	7106,60	8424,48	8435,30	10148,95
1,0667	1335,23	5987,59	1224,07	6235,72	1172,86	6768,81	1068,55	3347,02	5217,76	3218,62	4257,77	5153,45	6519,83	6877,31	8217,75	8231,99	9845,66
1,0700	1281,03	5821,78	1179,10	6042,02	1139,62	6581,47	1044,24	3232,28	4851,11	3115,60	4128,49	5013,51	6258,28	6649,44	7952,78	7974,76	9559,18
1,0733	1235,11	5672,52	1141,38	5868,49	1122,66	6404,26	1031,17	3111,82	4550,27	3062,76	4013,96	4858,67	6047,71	6449,66	7737,29	7727,36	9270,97
1,0767	1195,79	5536,03	1112,00	5696,44	1105,60	6226,92	1020,76	3000,23	4293,04	2981,92	3901,37	4726,91	5840,19	6251,59	7558,38	7553,32	8978,72
1,0800	1164,63	5412,06	1094,04	5541,66	1076,15	6074,77	1003,18	2899,50	4065,89	2906,21	3806,36	4624,87	5640,48	6073,77	7393,48	7353,84	8785,58
1,0833	1139,04	5292,92	1071,34	5390,19	1049,56	5940,32	1009,14	2806,82	3898,27	2859,44	3689,81	4476,61	5456,62	5892,64	7235,13	7112,21	8523,72
1,0867	1113,17	5184,01	1044,38	5253,68	1030,49	5801,29	990,43	2708,55	3716,50	2792,45	3595,20	4389,17	5290,62	5761,45	7037,93	6900,20	8328,12
1,0900	1089,11	5084,29	1021,59	5128,32	1014,77	5675,24	963,41	2620,37	3576,91	2734,36	3521,04	4284,23	5161,27	5621,63	6846,33	6738,34	8140,82
1,0933	1068,35	4987,77	1000,81	5012,98	1003,02	5559,62	941,56	2527,96	3464,33	2694,54	3436,00	4170,33	5029,62	5479,11	6652,02	6594,45	7965,39
1,0967	1049,87	4893,15	992,54	4907,24	992,67	5445,92	930,56	2450,20	3351,06	2641,26	3355,94	4079,24	4910,27	5339,97	6460,72	6426,76	7798,29
1,1000	1031,80	4807,78	980,11	4803,12	986,19	5345,81	915,15	2371,01	3243,34	2570,17	3300,98	3980,88	4783,34	5216,33	6280,90	6251,28	7621,34
1,1033	1016,13	4736,71	964,44	4711,61	974,35	5262,79	914,74	2288,75	3159,02	2518,57	3241,48	3876,81	4651,23	5111,72	6146,22	6102,36	7443,78
1,1067	1002,71	4669,16	953,06	4624,47	967,91	5181,17	914,97	2221,80	3076,36	2475,01	3214,18	3793,57	4547,90	5000,38	5994,13	5992,21	7297,57
1,1100	991,34	4605,55	936,80	4553,52	953,65	5102,43	905,44	2163,01	3008,97	2420,90	3150,60	3733,78	4456,07	4900,69	5835,03	5866,53	7134,89
1,1133	980,43	4547,49	926,91	4469,09	950,22	5029,42	892,77	2119,84	2951,07	2403,88	3107,00	3666,52	4344,11	4765,58	5718,89	5757,99	6984,02
1,1167	967,43	4486,11	919,96	4393,78	940,13	4958,19	896,24	2079,75	2916,99	2368,55	3088,50	3595,63	4254,38	4656,71	5614,21	5664,19	6858,19
1,1200	954,75	4418,26	907,94	4327,02	925,73	4896,60	887,30	2039,57	2860,06	2338,86	3055,62	3548,07	4144,94	4569,05	5530,95	5570,16	6738,55
1,1233	944,02	4362,53	895,76	4264,11	915,89	4836,30	876,59	1994,05	2851,29	2304,56	3013,48	3482,79	4054,04	4480,19	5455,81	5473,17	6622,71
1,1267	931,57	4321,60	890,72	4211,84	914,21	4780,96	867,84	1951,72	2830,06	2275,07	3001,21	3450,36	3989,24	4384,92	5388,30	5372,27	6502,07
1,1300	918,96	4279,36	882,19	4155,60	911,20	4727,67	870,08	1949,02	2789,57	2254,31	2991,51	3437,03	3919,90	4300,17	5324,69	5282,38	6402,66
1,1333	908,39	4232,61	871,10	4113,39	903,16	4686,30	873,28	1930,61	2764,07	2227,40	2964,62	3394,03	3847,52	4234,47	5293,50	5199,66	6325,78
1,1367	900,41	4194,33	866,70	4065,60	905,00	4650,50	862,77	1918,46	2751,36	2176,29	2953,86	3366,21	3786,41	4178,63	5272,77	5133,53	6237,52
1,1400	893,89	4158,12	864,29	4026,60	899,50	4610,36	865,96	1934,00	2725,46	2167,45	2941,21	3330,63	3720,71	4106,79	5299,09	5059,10	6150,12
1,1433	888,63	4120,09	857,28	3994,67	894,38	4570,38	863,32	1933,59	2693,23	2159,57	2933,52	3304,39	3652,42	4069,00	5283,28	4985,24	6088,02
1,1467	884,53	4087,20	859,28	3959,65	894,70	4536,12	872,34	1921,16	2661,54	2127,68	2939,98	3272,28	3619,43	4044,22	5281,15	4912,06	6052,45
1,1500	878,11	4057,58	860,32	3934,61	892,72	4513,60	873,75	1910,12	2649,27	2107,77	2930,75	3263,37	3583,98	4015,02	5280,30	4865,65	5991,06
1,1533	871,71	4034,09	848,61	3903,46	880,51	4494,76	861,81	1918,84	2638,04	2111,05	2908,52	3248,76	3525,60	3980,59	5285,85	4802,18	5956,04
1,1567	868,39	4014,32	849,59	3877,45	877,62	4469,23	856,32	1928,08	2621,23	2106,22	2907,63	3234,42	3496,40	3956,92	5300,45	4773,05	5908,24
1,1600	865,97	3999,87	850,66	3846,12	881,98	4450,62	843,96	1928,52	2584,30	2094,50	2919,27	3253,60	3487,01	3952,59	5321,24	4738,13	5872,19
1,1633	863,95	3984,78	851,51	3837,08	888,91	4431,42	847,03	1928,19	2550,49	2091,77	2933,48	3250,90	3474,57	3941,42	5328,14	4664,66	5839,47
1,1667	861,03	3973,87	843,65	3836,10	888,56	4431,13	845,66	1937,53	2521,04	2113,20	2942,65	3242,92	3461,28	3944,13	5308,11	4629,09	5819,33
1,1700	860,58	3962,92	841,87	3812,18	881,92	4427,55	849,07	1936,11	2516,52	2120,74	2956,27	3266,15	3476,30	3945,24	5239,03	4613,88	5828,08
1,1733	860,52	3953,43	854,64	3792,42	887,03	4422,46	847,87	1973,32	2472,85	2121,72	2961,82	3288,14	3477,49	3984,25	5201,24	4575,32	5831,33
1,1767	861,57	3942,85	856,44	3790,11	890,99	4417,10	857,38	1988,17	2443,11	2142,41	3004,98	3280,91	3479,31	3989,94	5167,79	4570,79	5803,09
1,1800	863,20	3935,66	849,39	3785,70	892,94	4412,63	853,49	1996,63	2408,29	2174,33	3034,39	3316,15	3510,45	3976,43	5116,95	4555,97	5815,88
1,1833	863,20	3930,43	857,42	3782,92	880,47	4424,06	862,10	2036,52	2373,46	2197,60	3047,65	3347,60	3539,15	4002,05	5066,13	4553,56	5814,43
1,1867	862,32	3929,66	863,32	3765,19	881,34	4441,47	858,34	2065,99	2351,96	2228,11	3088,75	3371,32	3562,88	4021,28	5031,46	4541,82	5827,29
1,1900	861,83	3932,20	862,30	3764,89	894,24	4452,12	853,99	2077,65	2333,58	2254,40	3141,63	3405,71	3588,12	4062,55	5011,32	4548,35	5845,75
1,1933	866,38	3940,57	854,25	3775,68	901,92	4446,34	865,45	2114,02	2322,87	2313,79	3179,59	3440,70	3638,52	4091,23	4983,69	4567,44	5883,89
1,1967	871,83	3948,78	854,62	3778,02	900,28	4462,66	852,92	2148,93	2304,73	2351,55	3239,68	3484,49	3677,05	4150,00	4986,47	4577,78	5915,76
1,2000	877,16	3964,93	865,43	3784,51	903,38	4497,80	873,39	2194,47	2303,52	2404,55	3267,31	3527,31	3734,59	4179,63	5000,60	4611,93	5937,13
1,2033	882,63	3984,53	877,99	3803,11	910,54	4522,50	889,43	2231,39	2290,60	2464,23	3310,84	3591,25	3766,54	4232,92	5028,03	4640,29	5991,34
1,2067	888,17	4006,59	879,85	3815,55	917,57	4534,18	886,88	2282,65	2291,72	2499,29	3394,99	3640,19	3840,58	4262,76	5058,39	4654,36	6049,92
1,2100	894,44	4034,05	886,68	3829,20	922,66	4561,73	877,90	2311,64	2282,44	2554,72	3441,19	3682,56	3921,82	4320,23	5071,45	4681,59	6085,68
1,2133	902,10	4053,73	877,92	3844,62	916,49	4597,92	878,29	2347,11	2293,31	2620,28	3490,68	3748,22	3985,45	4412,46	5139,26	4714,43	6128,60
1,2167	911,03	4076,96	874,78	3872,39	927,07	4631,85	872,50	2379,24	2304,82	2677,13	3559,53	3812,18	4051,77	4462,89	5203,39	4731,94	6182,92
1,2200	918,08	4107,73	882,75	3894,53	924,81	4674,53	871,29	2424,18	2315,39	2746,51	3603,89	3903,22	4120,82	4530,04	5263,40	4760,48	6236,24
1,2233	924,95	4141,04	893,89	3919,48	927,28	4717,56	886,16	2490,09	2328,02	2825,08	3650,02	4007,89	4202,63	4620,52	5337,20	4815,36	6274,96
1,2267	930,64	4174,89	902,67	3959,46	935,66	4747,96	903,24	2537,48	2371,03	2876,46	3726,31	4080,86	4287,07	4709,99	5433,69	4829,00	6323,27
1,2300	937,20	4218,66	900,13	3997,62	951,38	4771,11	906,18	2561,38	2417,29	2938,24	3779,64	4161,45	4393,89	4787,82	5519,20	4871,31	6389,20
1,2333	946,45	4261,75	914,86	4019,97	963,63	4807,22	917,13	2615,98	2448,19	3028,18	3878,25	4301,40	4484,55	4858,26	5628,74	4905,89	6459,40
1,2367	956,66	4310,79	927,09	4070,74	971,18	4868,40	918,34	2664,24	2500,92	3107,29	3948,18	4412,77	4598,58	4934,66	5739,45	4945,94	6529,29
1,2400	965,99	4358,29	927,58	4132,21	982,78	4927,20	928,31	2718,85	2546,79	3190,91	4039,27	4510,20	4695,71	5018,92	5811,19	4994,87	6579,30
1,2433	975,53	4409,35	932,13	4191,95	995,57	4987,21	941,54	2761,82	2605,08	3277,52	4149,27	4628,23	4804,59	5106,68	5917,05	5074,47	6670,29
1,2467	984,98	4469,54	949,77	4231,41	999,25	5037,40	955,77	2782,79	2678,82	3365,26	4235,27	4760,43	4923,69	5206,91	6004,70	5148,01	6781,50
1,2500	993,95	4535,47	951,44	4293,92	1002,82	5100,38	967,77	2843,69	2765,72	3440,06	4350,90	4891,45	5011,49	5318,03	6156,40	5216,65	6878,87
1,2533	1005,41	4608,84	961,38	4375,62	1024,75	5184,02	971,87	2914,93	2846,92	3554,12	4458,16	5028,51	5105,75	5441,98	6291,97	5301,47	6979,19
1,2567	1021,03	4688,17	968,87	4444,08	1049,27	5258,90	994,17	2968,59	2905,82	3667,82	4616,74	5163,54	5235,35	5535,92	6379,62	5379,95	7109,57
1,2600	1039,29	4768,12	986,35	4516,58	1067,50	5324,49	1012,18	3028,96	2996,27	3784,75	4765,96	5305,95	5345,50	5650,15	6515,35	5480,56	7267,97
1,2633	1060,44	4850,49	1001,50	4590,01	1086,38	5413,47	1030,47	3116,36	3081,18	3925,35	4918,67	5449,53	5432,85	5777,70	6677,73	5601,36	7453,42
1,2667	1084,00	4943,70	1017,81	4682,21	1110,57	5511,04	1045,82	3213,65	3148,63	4074,40	5089,85	5582,24	5535,08	5932,51	6842,87	5714,00	7653,49
1,2700	1112,02	5045,68	1045,60	4771,90	1134,11	5604,14	1074,62	3319,38	3256,97	4234,52	5285,47	5799,18	5650,78	6063,22	6976,34	5844,78	7855,47
1,2733	1141,53	5158,61	1064,09	4876,52	1153,95	5691,56	1090,37	3448,40	3347,93	4416,24	5499,81	6003,88	5797,54	6211,27	7202,41	5983,20	8018,05
1,2767	1172,39	5280,65	1088,25	4989,63	1177,93	5797,62	1125,21	3602,23	3415,11	4583,13	5725,74	6171,32	5947,46	6375,87	7378,66	6096,92	8241,06
1,2800	1206,62	5409,54	1121,22	5095,23	1213,43	5915,34	1162,85	3753,86	3504,59	4745,57	5947,67	6371,92	6096,21	6546,01	7569,29	6260,76	8441,62
1,2833	1247,85	5553,11	1162,21	5216,07	1257,64	6038,12	1202,00	3893,16	3602,93	4891,14	6212,18	6634,83	6268,98	6697,72	7752,00	6410,34	8660,36
1,2867	1298,37	5713,39	1204,22	5352,71	1306,04	6172,90	1264,73	4064,63	3723,56	4979,00	6517,67	6856,88	6499,83	6935,32	8017,70	6575,31	8891,90
1,2900	1357,54	5876,99	1259,34	5510,33	1365,74	6326,80	1319,51	4208,99	3831,94	5049,30	6830,39	7049,46	6713,32	7180,17	8264,21	6738,31	9170,44
1,2933	1426,19	6060,05	1312,77	5675,94	1440,83	6497,04	1394,53	4312,84	3923,13	5072,99	7232,72	7303,63	6964,30	7411,66	8460,23	6957,98	9374,64
1,2967	1505,59	6259,49	1392,88	5859,83	1527,83	6684,13	1480,50	4446,23	4057,71	5141,08	7601,94	7543,57	7219,69	7639,58	8720,12	7144,93	9610,77
1,3000	1596,81	6467,29	1495,20	6066,08	1622,39	6889,80	1602,11	4601,23	4172,14	5273,55	7966,01	7781,63	7444,85	7900,83	9014,73	7339,04	9830,08
1,3033	1702,06	6687,07	1622,52	6271,70	1709,02	7107,56	1731,88	4833,65	4250,66	5544,44	8367,92	8001,67	7682,95	8162,25	9237,78	7576,11	10072,26
1,3067	1815,25	6916,30	1721,93	6458,30	1785,84	7352,03	1752,46	5201,06	4335,27	6010,87	8765,65	8258,66	7865,64	8403,17	9582,39	7810,12	10294,33
1,3100	1932,70	7184,28	1826,78	6661,64	1879,52	7596,01	1795,68	5651,11	4388,48	6623,19	9222,29	8765,51	8039,98	8719,88	9949,91	8047,62	10521,54
1,3133	2061,36	7528,35	1930,56	6882,52	1995,46	7848,38	1894,07	6515,93	4437,41	7428,97	9870,13	9335,20	8301,44	9026,51	10293,88	8304,41	10739,33
1,3167	2210,73	7949,94	2091,80	7134,86	2132,40	8137,90	2074,17	7435,07	4495,32	8848,88	10519,92	10029,58	8632,75	9345,53	10606,66	8599,90	10965,45
1,3200	2407,69	8494,43	2302,64	7406,18	2371,60	8445,67	2311,91	8763,28	4582,61	10479,05	11230,18	11046,28	9016,42	9550,63	11044,46	8945,31	11238,03
1,3233	2685,02	9207,20	2675,70	7792,11	2712,69	8758,36	2675,47	10658,27	4786,83	12608,51	12096,53	12365,58	9575,13	9852,45	11577,41	9281,31	11513,82
1,3267	3119,55	10082,89	3066,03	8252,36	3352,38	9146,38	2873,09	13074,77	5159,51	15532,42	13161,75	14183,36	10343,74	10331,23	12166,15	9623,68	11829,76
1,3300	3873,35	11107,21	4212,24	8994,78	4878,51	9651,63	3916,68	16196,28	5643,52	18778,83	14375,75	16198,37	11495,63	10795,61	12888,76	9982,79	12183,29
1,3333	5222,28	12354,67	5939,15	9881,30	6718,46	10285,49	6705,56	19488,55	6366,42	23224,16	15784,19	18524,04	12975,70	11377,59	13727,97	10370,26	12621,83
1,3367	7377,09	13800,32	8374,32	11086,51	10068,87	11098,24	10422,81	23563,66	7333,04	26745,70	17418,12	20829,41	14733,28	12231,44	14838,45	10820,16	13246,14
1,3400	10540,43	15431,15	11317,85	12421,52	14811,03	12174,67	15572,51	27050,57	8536,27	29665,80	19134,99	23465,91	17008,43	13301,90	16288,37	11216,42	13924,87
1,3433	14682,04	17214,28	15839,56	14219,19	19721,39	13615,73	21372,28	30357,51	9975,71	31292,66	21282,82	25483,32	19449,93	14877,67	17811,08	11735,77	14702,53
1,3467	19503,72	19148,88	20996,48	16157,49	25490,48	15359,16	28479,19	32361,56	11532,53	31483,50	23370,08	26938,14	22157,99	16716,91	19339,80	12302,79	15541,24
1,3500	24453,18	21199,12	25035,88	18261,48	29161,63	17463,50	33669,19	32721,15	13325,44	31876,34	25082,24	27792,89	24145,11	18924,24	20701,59	13063,26	16541,14
1,3533	28953,60	23202,19	30436,10	20701,94	32380,26	19455,52	37914,16	32981,57	15219,05	31280,74	26260,72	27957,91	25855,08	20927,50	21817,59	13871,84	17570,58
1,3567	32725,69	24963,39	33495,27	22765,19	35627,39	21680,73	39238,60	32411,08	17510,89	31524,14	26834,99	28169,57	27022,72	22748,43	22759,87	14798,20	18612,43
1,3600	35339,98	26337,06	36391,23	25100,76	36999,11	23675,56	37975,12	32713,62	19867,44	32123,62	27365,32	27962,79	27581,68	24039,71	23196,62	15898,92	19679,91
1,3633	36631,45	27232,48	37854,77	26577,67	37028,34	25351,25	36791,81	33253,12	22870,57	32839,22	27436,40	28101,90	28120,32	24590,63	23603,77	17020,97	20543,07
1,3667	36750,34	27658,42	36998,66	27699,10	36401,39	26538,30	33266,50	33664,23	25658,42	34489,68	27751,38	28614,27	28098,06	24975,98	23852,54	18040,25	21284,68
1,3700	36085,75	27822,83	36754,80	28298,49	34555,49	26971,53	32458,34	35183,93	28719,16	34970,91	28026,74	29151,88	28379,88	24705,18	24091,86	18662,33	21811,45
1,3733	35340,78	27827,04	34183,23	28092,95	35763,88	27040,00	33326,00	35979,62	30943,94	35092,45	28438,86	30110,35	28792,75	24792,74	24458,05	19278,10	22222,01
1,3767	34938,37	27952,26	33942,47	28146,66	36538,87	27168,70	34602,73	36495,08	31845,38	34807,30	29340,57	30538,86	29171,66	25052,23	24753,96	19704,55	22571,05
1,3800	35227,01	28255,29	34345,84	27795,25	36851,95	26958,04	38483,37	36362,30	32512,38	33617,17	29981,75	30723,31	29848,42	25450,08	25075,05	20042,32	22911,95
1,3833	36212,12	28670,22	34506,70	27982,28	39138,33	27117,74	40065,67	35381,23	32092,40	33533,04	30749,38	30761,91	30149,60	26480,65	25397,49	20368,00	23228,97
1,3867	37498,55	29194,82	37816,27	28535,50	40233,06	27526,23	40891,96	34994,43	32241,91	33150,39	30961,72	30246,89	30301,60	27066,74	25701,39	20527,27	23500,11
1,3900	38676,88	29629,32	39147,54	29009,21	40369,43	27889,63	40195,99	34385,59	32772,81	33310,56	30998,17	30030,92	30349,02	27410,53	26121,83	20786,70	23816,34
1,3933	39166,22	29833,64	39762,59	29878,58	39728,17	28661,78	37015,07	34300,84	33381,23	34523,46	30956,08	29880,15	30203,25	27596,35	26336,77	21012,66	24081,81
1,3967	38792,25	29822,57	39442,96	30237,72	37314,84	29146,87	35375,23	34963,48	34816,90	35262,21	30565,89	29912,69	30243,03	27146,63	26628,61	21189,10	24318,65
1,4000	37801,55	29622,23	36820,31	30321,35	35391,08	29203,19	33003,53	35358,08	35396,75	36424,63	30618,13	30624,85	30258,36	26957,73	26861,37	21488,31	24569,43
1,4033	36612,32	29464,25	36137,32	30314,36	36194,28	29147,82	32818,13	36454,60	35446,52	36508,98	30578,62	30962,54	30363,49	26939,36	27043,04	21680,06	24781,43
1,4067	35876,44	29424,03	34351,99	29745,93	36374,11	28848,87	35776,65	36868,44	35196,75	35846,12	30749,95	31385,37	30658,23	27086,10	27229,96	21797,89	24948,45
1,4100	35824,05	29570,70	34030,11	29626,48	37109,91	28459,48	37516,74	36511,71	34286,71	35358,49	31369,87	31532,63	30930,72	27911,42	27254,77	21888,11	25126,42
1,4133	36484,39	29928,70	36032,31	29522,86	39457,64	28493,34	40216,82	36058,04	34052,92	33934,89	31684,89	31123,09	31355,27	28474,41	27521,81	21879,39	25367,15
1,4167	37676,15	30292,57	36766,57	29679,20	39874,94	28660,58	40855,02	34980,43	34311,37	33762,72	32172,86	30747,64	31456,17	28895,42	27775,04	22049,58	25572,07
1,4200	38788,58	30610,34	39294,54	30309,02	40645,20	28848,16	39618,00	34671,09	34665,66	33653,88	32296,32	30252,32	31330,39	29024,50	27943,29	22167,73	25802,10
1,4233	39387,42	30766,11	40210,11	30641,26	40077,50	29537,35	38040,23	34415,10	35560,86	33812,91	32170,19	30116,36	31202,20	28614,57	27975,18	22268,69	25970,58
1,4267	39172,32	30679,40	39391,10	31167,29	37402,50	29908,36	34105,79	34461,38	35845,37	35406,90	32100,02	30263,08	30921,50	28522,01	28090,45	22474,34	26174,79
1,4300	38210,65	30468,96	38468,90	31303,92	36160,21	30059,67	33012,30	35345,37	35521,93	36128,80	31641,26	30463,88	30824,04	28329,95	28228,70	22525,62	26295,40
1,4333	37018,71	30199,89	35472,86	30964,09	36222,21	30054,21	33145,04	35641,02	35244,82	37021,79	31712,40	30949,56	30773,93	28328,53	28302,00	22735,02	26409,82
1,4367	36059,07	30022,42	34964,89	30758,29	36204,34	29661,73	34287,13	36069,67	34192,46	36900,67	31690,64	31180,13	30852,37	29034,63	28550,52	22915,08	26543,42
1,4400	35804,02	30046,26	34563,52	30188,22	37565,70	29422,20	38029,21	36003,90	33904,81	36011,54	31710,44	31496,95	31164,13	29494,97	28693,95	23049,55	26646,10
1,4433	36318,96	30273,86	34482,42	30141,06	39273,60	29328,11	39603,62	35381,02	33953,32	35500,87	32376,76	31651,63	31491,47	29917,53	28763,33	23212,68	26819,24
1,4467	37324,61	30674,02	37581,90	30369,13	39362,28	29205,91	41038,00	35143,02	34087,13	34407,67	32873,27	31641,60	31963,72	29962,40	28834,65	23407,60	26899,19
1,4500	38483,13	31022,43	38727,54	30562,54	40629,41	29451,03	40652,50	34696,02	35069,23	34392,30	33460,02	31669,36	32119,61	29324,28	28850,30	23615,81	27144,27
1,4533	39157,38	31193,21	39918,77	31186,14	39967,67	30038,03	37789,39	34687,48	35516,54	34156,93	33626,89	31395,64	32061,72	28960,53	28882,91	23827,29	27328,70
1,4567	39058,49	31147,02	39981,25	31419,82	37584,52	30232,00	36105,34	35238,43	35986,42	34169,39	33459,60	31295,71	32037,44	28494,78	28877,69	24093,78	27408,30
1,4600	38243,50	30868,64	37921,37	31582,08	36890,44	30393,59	33217,59	35610,15	35981,21	35504,65	33220,99	31437,47	31883,17	28263,32	28839,34	24441,50	27465,09
1,4633	37048,16	30536,28	37040,18	31548,52	36027,20	30321,32	32840,65	36387,66	35471,18	36231,93	32670,69	31554,74	31937,94	28663,68	28798,29	24722,09	27545,71

1,4667	36124,75	30337,18	34693,32	30958,70	36200,52	29863,58	35077,38	36627,63	35231,85	37284,71	32611,68	32204,67	32003,85	28778,16	28697,72	25020,22	27653,99
1,4700	35776,69	30303,62	34347,11	30697,72	37794,17	29698,65	37084,71	36340,16	34490,76	37500,18	32315,41	32491,22	32049,31	28863,66	28667,72	25144,18	27829,34
1,4733	36155,76	30495,31	35638,41	30320,47	38653,64	29451,36	40233,88	36012,63	34234,01	36995,64	32241,84	32352,91	32206,71	28584,07	28618,33	25207,55	27911,29
1,4767	37182,94	30799,19	36061,13	30377,01	39188,20	29286,51	41009,06	35310,94	34586,23	36485,56	32814,99	32108,94	32263,36	28151,66	28600,92	25362,60	27917,20
1,4800	38370,65	31132,81	38939,93	30867,99	40717,48	29595,17	40511,28	35136,68	34845,52	35245,70	33156,08	31529,97	32298,84	27864,97	28576,57	25307,47	27912,55
1,4833	39279,70	31400,97	40090,48	31102,53	40004,41	30015,74	39312,46	35357,07	35830,62	35119,94	33909,09	31361,49	32320,09	27523,18	28582,88	25373,30	27930,76
1,4867	39444,86	31439,31	39938,59	31622,47	38230,12	30308,21	35493,39	35664,29	36145,79	34887,71	34111,43	31624,01	32308,24	27379,89	28514,44	25446,92	27906,96
1,4900	38799,87	31275,97	39177,89	31788,54	37480,62	30611,74	33935,65	36718,13	36274,38	34984,05	34035,54	31778,85	32271,18	27506,96	28483,51	25497,04	27851,68
1,4933	37736,21	30929,72	36250,54	31565,72	35776,06	30533,60	33151,77	37132,73	36355,84	36024,12	33796,54	32480,04	32319,21	27404,03	28361,70	25710,26	27814,99
1,4967	36696,08	30583,64	35757,51	31335,24	36327,23	30136,51	33884,04	37462,18	35781,54	36490,99	33153,49	32627,64	32422,47	27348,16	28254,42	25884,64	27803,75
1,5000	36194,48	30455,57	34703,09	30657,19	37696,30	29964,49	37573,75	37423,74	35556,98	37392,59	32961,27	32485,59	32666,53	27235,29	28150,52	26088,87	27836,39
1,5033	36386,41	30571,86	34385,49	30440,63	37986,65	29562,63	39416,88	36449,63	34980,17	37319,28	32470,95	32154,22	32777,19	26989,81	27964,10	26245,17	27808,09
1,5067	37185,38	30918,43	36984,90	30386,89	39442,98	29408,83	41259,13	35880,56	34738,84	36463,12	32288,82	31331,14	32673,06	26850,43	27676,09	26429,41	27734,04
1,5100	38352,11	31281,85	37961,21	30516,63	40915,72	29696,98	41143,71	35134,18	35287,80	35872,23	32643,43	31006,93	32577,08	26733,79	27435,34	26665,05	27734,55
1,5133	39283,29	31518,10	39736,92	31143,71	40199,50	29945,76	38810,70	35111,59	35730,96	34776,44	32777,19	30833,54	32353,55	26584,57	27194,02	26767,41	27760,27
1,5167	39593,56	31578,18	40206,94	31395,36	38882,91	30348,79	37049,29	35734,04	36891,32	34636,61	33621,51	30799,03	32263,12	26525,13	27120,97	26949,61	27664,31
1,5200	39128,34	31399,88	38734,48	31696,26	37491,79	30700,80	33602,12	36056,40	37550,57	34937,30	34042,17	31484,39	32100,92	26688,43	27120,19	27005,06	27568,52
1,5233	38091,68	31085,31	37775,16	31673,53	35500,03	30611,76	32647,14	37045,60	37725,43	35196,91	34250,71	31785,28	32172,80	26810,61	27197,88	27104,65	27582,44
1,5267	37061,98	30753,22	35053,35	31024,64	36547,52	30339,10	34358,59	37252,57	37619,77	36447,88	34079,43	32260,69	32311,78	26818,95	27533,87	27162,90	27517,54
1,5300	36405,77	30597,33	34771,14	30755,51	37507,34	30102,86	36285,78	36886,42	36740,99	36797,87	33413,65	32320,01	32448,26	26818,02	27807,05	27398,81	27479,07
1,5333	36432,15	30717,05	35319,86	30259,93	37712,54	29623,48	39767,89	36398,58	36337,99	36773,88	32969,87	31874,77	32661,52	26874,00	28261,90	27623,65	27445,66
1,5367	37153,79	30991,69	35472,07	30206,36	39797,43	29474,57	40886,36	35347,07	35766,71	36337,94	32167,81	31552,24	32708,30	26795,81	28775,08	27768,87	27284,82
1,5400	38211,81	31305,34	38519,38	30475,10	40794,76	29612,86	40934,22	34963,59	35580,94	35351,43	31981,74	30750,63	32744,78	26730,38	29112,65	28000,49	27093,53
1,5433	39229,29	31524,94	39623,67	30672,56	40537,31	29711,08	39814,94	34876,93	35950,29	35020,39	31978,22	30472,20	32566,36	26701,75	29492,94	28122,67	26959,66
1,5467	39656,27	31540,45	40024,59	31238,60	39580,14	30243,37	36115,71	35028,68	35958,93	34469,75	32103,21	30664,95	32249,38	26644,85	29769,49	27910,58	26713,57
1,5500	39290,29	31355,12	39612,63	31428,89	37283,45	30567,08	34325,21	35941,02	36614,84	34655,54	32965,23	30969,96	31981,93	26723,80	30007,32	27942,19	26510,83
1,5533	38332,15	31002,96	37002,80	31348,73	35577,96	30480,82	32865,14	36320,93	36753,84	35416,93	33575,98	31862,30	31772,18	26796,91	30132,08	27767,16	26292,21
1,5567	37165,92	30679,76	36378,72	31126,36	36552,20	30247,92	33172,41	36615,48	36447,34	35739,46	34241,26	32247,03	31693,06	26875,92	30201,51	27828,82	25990,90
1,5600	36395,27	30519,09	34775,92	30383,99	37031,44	29750,53	36436,37	36502,63	36122,41	36804,51	34318,57	32294,53	31660,52	26849,70	30396,62	28069,77	25781,81
1,5633	36253,26	30566,27	34457,01	30142,68	37649,68	29205,32	38392,42	35683,82	35178,71	37036,34	33868,43	32061,86	31561,52	26722,29	30543,61	28027,73	25536,54
1,5667	36771,04	30830,99	36409,75	29939,46	39992,58	29076,54	40642,91	35280,76	34822,44	36566,09	33345,51	31122,68	31656,82	26694,95	30806,68	28349,33	25170,55
1,5700	37809,45	31150,67	37076,07	29989,42	40455,08	29025,41	40833,25	34813,08	34810,88	35996,52	32381,15	30622,13	31564,25	26496,07	30948,27	28444,97	24841,96
1,5733	38838,36	31362,03	39387,58	30565,19	40703,36	29103,21	38948,62	34747,49	34911,27	34845,94	32111,10	30248,38	31589,57	26382,82	31134,90	28238,60	24393,71
1,5767	39462,52	31379,38	40252,11	30754,68	39751,67	29711,82	37036,09	35326,00	35655,09	34495,88	31704,22	30168,92	31515,91	26303,35	31192,37	28234,77	24043,44
1,5800	39337,39	31148,14	39380,54	31126,30	37028,45	30031,11	33352,45	35568,00	35873,31	34058,22	31564,35	30732,05	31429,82	26174,31	30893,57	27884,45	23704,27
1,5833	38508,07	30799,98	38496,31	31161,46	36042,16	30075,92	32450,52	36080,04	35930,82	34180,70	32067,21	30927,41	31309,90	26111,60	30654,65	27799,92	23411,28
1,5867	37449,88	30468,53	35653,93	30689,88	36519,28	29954,62	33562,71	36089,81	35877,84	35411,96	32353,26	31111,63	31141,29	26066,78	30401,61	27989,71	23169,96
1,5900	36576,86	30276,18	35298,08	30378,85	36758,68	29468,99	35250,13	35242,33	35326,33	35859,88	33241,96	30998,94	31024,07	25827,90	30240,54	27935,06	22958,81
1,5933	36316,44	30268,23	35090,03	29749,91	38055,95	29103,97	38953,79	34654,54	34851,07	36449,31	33646,63	30258,30	30948,73	25618,66	30303,53	28313,74	22794,50
1,5967	36745,75	30399,45	34928,04	29641,24	39888,44	28894,94	40511,07	33811,54	34453,40	36192,38	33545,88	29855,16	31028,99	25468,78	30156,71	28607,32	22687,95
1,6000	37642,18	30642,23	37826,28	29832,00	39968,38	28665,88	41200,39	33509,90	34310,65	35021,51	33298,84	29319,72	31227,73	25308,47	29962,11	28792,61	22553,10
1,6033	38708,30	30835,76	38893,68	29850,32	40715,80	28757,57	40282,60	33789,79	34658,86	34515,48	32603,68	29119,75	31461,93	25296,22	29598,53	28631,37	22483,45
1,6067	39382,16	30846,94	39928,66	30380,27	39650,77	29229,03	36916,68	33924,52	34859,96	33741,82	32112,64	29293,14	31692,72	25269,35	29358,61	28092,15	22417,10
1,6100	39380,70	30652,51	39875,06	30427,54	36971,42	29397,09	34976,25	34746,71	35203,24	33710,93	31330,01	29391,02	31760,75	25038,69	29226,12	27997,67	22225,28
1,6133	38709,16	30257,46	37769,27	30337,38	36533,31	29480,77	32783,05	34924,61	35133,38	34651,89	31076,63	29857,74	31751,24	24717,04	29146,63	27819,70	22036,39
1,6167	37649,35	29811,20	37126,05	30121,36	36165,19	29282,79	32815,76	34746,91	34618,32	35055,91	30840,75	29993,08	31719,83	24354,67	29271,45	27716,45	21868,39
1,6200	36779,83	29459,70	35121,54	29343,28	36454,91	28758,96	35783,43	34337,79	34079,27	35677,55	30722,03	30163,82	31723,56	23993,97	29288,13	27941,84	21738,23
1,6233	36383,79	29314,11	34705,59	29042,46	38116,17	28433,16	37899,36	33171,71	33495,21	35410,28	31170,71	30014,39	31786,67	23749,43	29225,98	28116,65	21597,56
1,6267	36622,77	29392,12	35824,95	28693,27	39216,21	28071,62	40652,98	32570,97	33056,29	34478,34	31382,60	29547,13	31827,47	23575,43	29303,28	28370,92	21442,40
1,6300	37429,54	29566,23	36076,52	28577,09	39668,18	27760,25	41129,98	32015,61	32867,02	33941,94	31821,96	29116,56	31845,41	23293,95	29253,45	28432,81	21330,47
1,6333	38380,02	29707,62	38678,34	28881,93	40652,02	27837,53	40065,45	31731,07	32237,28	32872,07	31905,68	28366,29	31872,50	22971,46	29150,71	28226,31	21197,01
1,6367	39110,10	29650,97	39745,21	28837,15	39391,36	28011,36	38516,32	32268,97	31126,42	32483,29	31723,83	27996,29	31902,21	22459,61	29017,22	28210,26	20988,03
1,6400	39187,85	29331,30	39506,93	29091,81	37127,62	28074,97	34425,36	32266,51	29991,92	32862,98	31410,47	27816,88	31914,38	21969,32	28781,29	28034,67	20927,21
1,6433	38554,95	28795,60	38730,13	28970,90	36615,02	28109,03	32714,01	32378,91	28683,88	32935,62	30783,38	27669,28	31869,91	21313,59	28470,92	27927,42	20758,47
1,6467	37559,22	28140,46	35822,73	28323,64	35590,09	27729,97	32547,52	31794,75	27158,30	33913,99	30384,98	28195,96	31733,22	20823,83	28090,11	27948,96	20570,21
1,6500	36549,10	27587,42	35325,85	27806,45	36324,76	27106,73	33499,13	30200,44	25404,68	33983,07	29744,22	28235,63	31570,54	20315,80	27794,40	27696,39	20386,06
1,6533	35966,27	27237,11	34393,62	26926,42	37575,37	26646,88	36981,80	28256,52	23413,07	33656,86	29413,25	27918,00	31505,88	19670,95	27417,25	27608,34	20187,51
1,6567	35879,29	26986,81	33766,80	26519,21	37030,43	26009,87	38777,33	25337,85	21041,28	32888,23	29302,23	26889,69	31448,34	19157,46	26829,59	27559,52	19974,02
1,6600	36094,99	26738,42	35744,59	26306,34	37129,92	25560,39	40397,65	22424,27	18817,68	30452,41	29113,47	24623,13	31526,84	18528,74	26362,87	27181,58	19772,89
1,6633	36313,08	26358,29	35396,02	25965,75	37287,09	25446,29	40477,45	19160,15	16868,99	27841,92	29379,95	22250,57	31691,73	17585,49	25783,41	26817,85	19561,50
1,6667	35932,66	25725,25	35697,67	26140,61	35631,52	25226,39	37753,10	15995,60	15392,97	23364,03	29465,88	19075,57	31737,71	16556,22	24986,47	26538,47	19277,67
1,6700	34657,50	24589,76	34282,17	25679,64	32851,20	25103,06	33670,45	13648,38	14516,54	18696,25	29593,23	16142,75	31610,09	15241,85	23754,06	26479,42	19133,23
1,6733	32446,18	22876,56	31058,00	25044,80	28549,20	24801,30	25539,96	11800,22	13905,92	14905,55	29579,16	13945,24	31389,93	14033,04	22021,62	26456,89	18999,13
