# Supplementary material for: Photographic-Based Optical Evaluation of Tissues and Biomaterials Used for Corneal Surface Repair: A New Easy-Applied Method
Source: PLoS One. 2015 Nov 13;10(11):e0142099. doi: 10.1371/journal.pone.0142099 (PMC4643926; doi:10.1371/journal.pone.0142099)
Supplement: S2 Appendix — (PDF) [file pone.0142099.s002.pdf]

PIXELS	PETRI A	PCCS A	PETRI B	PCCS B	PETRI C	PCCS C	PETRI AM	1LAYER-AM A	1LAYER-AM B	1LAYER-AM C	2LAYER-AM A	2LAYER-AM B	2LAYER-AM C	3LAYER-AM A	3LAYER-AM B	4LAYER-AM A	4LAYER-AM B
0,6467	2500,69	8042,34	2408,58	7797,70	2363,63	8582,07	2285,91	9700,58	4584,44	7701,47	14047,70	11610,38	8560,13	8905,48	10997,78	9000,73	10757,22
0,6500	2782,18	8538,34	2720,79	8249,03	2623,49	9055,50	2474,46	11618,03	4762,33	9061,12	15788,12	13294,20	8817,25	9140,89	11422,18	9333,42	11102,08
0,6533	3210,32	9163,87	3262,72	8808,28	3094,70	9594,83	2876,15	14440,47	4957,12	11296,75	17995,69	15012,39	9122,87	9418,40	11927,64	9677,42	11446,97
0,6567	3971,12	10019,52	4208,35	9504,07	3844,51	10281,88	3494,00	17476,76	5277,66	15470,30	20215,78	17264,94	9647,08	9767,52	12565,06	10033,83	11925,46
0,6600	5466,73	11149,48	6614,21	10574,19	6158,89	11271,40	4649,19	21458,01	5811,92	19735,42	22926,68	19393,40	10314,52	10131,13	13372,82	10485,39	12417,41
0,6633	8047,90	12535,09	9295,27	11769,76	10278,02	12744,26	5724,71	24825,39	6663,84	25249,86	24976,05	21749,82	11197,49	10747,68	14427,34	10879,08	12977,14
0,6667	11952,23	14221,21	14155,96	13459,65	15366,92	14701,58	7965,13	27717,20	7615,92	29052,38	26253,88	23811,49	12190,74	11572,30	15513,51	11318,92	13744,70
0,6700	17018,07	16216,85	20066,53	15370,53	21491,70	16802,19	13565,36	29912,18	8848,79	30806,50	27232,65	25145,55	13312,53	12681,48	16989,25	11773,17	14511,94
0,6733	22682,19	18453,04	25061,35	17675,43	27437,65	19067,22	19759,70	30620,63	10502,85	31687,94	27672,42	26190,57	14663,53	13914,90	18479,34	12405,11	15433,01
0,6767	28219,60	20816,52	31285,69	20255,71	30764,28	21386,22	28415,36	31137,41	12561,76	31141,61	28389,32	26693,98	16170,63	15323,88	20114,22	13125,79	16489,65
0,6800	32828,90	23134,88	35394,06	22585,85	35549,80	23465,97	33888,73	31066,45	15161,89	31853,59	28681,50	27301,07	17781,18	17169,63	21649,16	14121,82	17581,85
0,6833	36052,53	25142,51	38543,84	25163,57	38297,80	25491,92	36148,75	31479,06	17677,22	31861,15	29221,50	27479,75	19690,08	19080,82	22902,92	15254,22	18609,17
0,6867	37489,68	26732,85	39509,64	26748,16	38740,86	26601,15	36560,21	32090,18	20419,32	32208,71	30077,63	27860,88	21606,64	21114,42	23798,55	16601,07	19585,43
0,9900	37292,48	27997,31	37569,23	28177,21	38297,50	26237,72	40629,18	22611,64	33725,04	20616,19	18725,32	17551,98	29944,39	26763,35	24574,30	20229,29	22966,56
0,9933	36870,18	27674,30	36511,75	27953,10	37925,75	26410,00	36399,85	18942,42	33738,16	16058,96	15675,51	14914,74	29062,39	26348,26	23614,38	20004,41	22428,19
0,9967	34915,22	26837,54	32669,49	27643,80	36106,87	25978,17	31200,92	15528,45	33987,31	12026,44	13507,13	12690,64	27911,41	25094,14	22477,45	19467,21	21909,16
1,0000	31662,89	25392,33	29077,09	26571,59	32479,51	25340,51	21856,67	12515,22	33937,77	9629,69	11780,59	11330,76	26048,69	23707,11	20763,02	18979,92	21252,85
1,0033	27677,98	23399,86	23301,70	24627,02	27099,59	24216,57	12267,72	10637,18	33399,32	8148,79	10663,22	10335,94	24164,72	21238,71	19184,35	18300,79	20403,75
1,0067	23464,97	21067,78	18880,98	22456,18	23319,50	22400,03	6060,81	9262,99	32794,77	7485,36	9833,73	9685,03	21790,18	18862,90	17457,07	17359,57	19683,44
1,0100	19076,27	18685,46	13036,20	19774,79	18778,73	20846,93	3280,87	8475,65	31013,41	6938,28	9275,63	9182,17	19478,52	16432,88	15929,76	16142,35	18789,53
1,0133	14739,38	16375,43	8248,12	17481,50	12013,13	18815,02	3014,56	7866,15	28979,72	6541,17	8746,38	8789,38	17357,02	14260,08	14739,71	14788,95	17799,94
1,0167	10836,42	14261,08	5804,19	15173,02	7156,57	16705,02	2556,84	7423,50	25870,58	6210,93	8242,03	8401,54	15345,90	13003,69	13689,58	13780,83	16800,69
1,0200	7760,05	12491,93	3926,19	13309,18	4750,82	14889,94	2299,92	6994,04	22370,07	5867,28	7723,06	8007,82	13620,54	12021,61	12933,96	12768,70	16016,66
1,0233	5632,14	11119,50	3324,42	11758,52	2653,53	13229,48	2147,90	6515,03	18522,48	5456,38	7254,81	7630,91	12125,57	11255,25	12297,13	11944,41	15120,97
1,0267	4259,48	10111,19	2760,13	10573,37	2513,88	11847,00	2015,46	6081,17	14974,08	5041,73	6791,17	7290,04	11117,76	10616,80	11703,58	11199,14	14389,18
1,0300	3431,73	9357,14	2412,18	9729,61	2199,63	10882,68	1904,83	5632,93	12227,81	4700,46	6393,77	6962,21	10301,35	10172,22	11155,34	10671,07	13736,82
